# Multiomics of early epileptogenesis in mice reveals phosphorylation and dephosphorylation-directed growth and synaptic weakening

**DOI:** 10.1016/j.isci.2024.109534

**Published:** 2024-03-19

**Authors:** Mariella Hurtado Silva, Ashley J. van Waardenberg, Aya Mostafa, Susanne Schoch, Dirk Dietrich, Mark E. Graham

**Affiliations:** 1 Synapse Proteomics, Children’s Medical Research Institute, The University of Sydney, Westmead, NSW 2145, Australia; 2 i-Synapse, Cairns, QLD 4870, Australia; 3 Department of Neuropathology, University Hospital Bonn, Synaptic Neuroscience Unit, 53127 Bonn, North Rhine-Westphalia, Germany; 4 Department of Neurosurgery, University Hospital Bonn, Synaptic Neuroscience Unit, 53127 Bonn, North Rhine-Westphalia, Germany

**Keywords:** Molecular biology, Neuroscience, Omics, Proteomics

## Abstract

To investigate the phosphorylation-based signaling and protein changes occurring early in epileptogenesis, the hippocampi of mice treated with pilocarpine were examined by quantitative mass spectrometry at 4 and 24 h post-status epilepticus at vast depth. Hundreds of posttranscriptional regulatory proteins were the major early targets of increased phosphorylation. At 24 h, many protein level changes were detected and the phosphoproteome continued to be perturbed. The major targets of decreased phosphorylation at 4 and 24 h were a subset of postsynaptic density scaffold proteins, ion channels, and neurotransmitter receptors. Many proteins targeted by dephosphorylation at 4 h also had decreased protein abundance at 24 h, indicating a phosphatase-mediated weakening of synapses. Increased translation was indicated by protein changes at 24 h. These observations, and many additional indicators within this multiomic resource, suggest that early epileptogenesis is characterized by signaling that stimulates both growth and a homeostatic response that weakens excitability.

## Highlights

• Postsynaptic density proteins closest to the membrane layer were dephosphorylated
• Phosphorylation and protein changes indicated homeostatic synaptic weakening
• Posttranscriptional regulatory proteins were the major targets of phosphorylation
• Predicted upstream protein kinases were known regulators of plasticity and growth

## Graphical abstract

**Figure:**
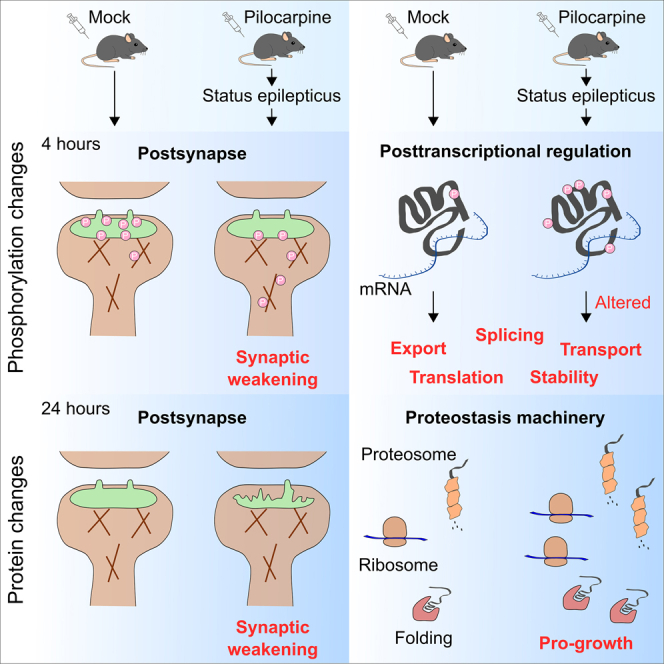

## Introduction

Epileptogenesis constitutes the process by which brain tissue becomes capable of generating spontaneous recurrent seizures^1^ and is triggered by genetic factors or a precipitating insult, followed by a latent period before the first observation of a clinical seizure. In experimental animal models of epileptogenesis, induction of status epilepticus (SE) results in chronic recurring seizures after a latent period.^2^ The identification of factors involved in the development of chronic epilepsy may form the basis for the discovery of new anti-epileptogenesis drugs.

A persistent hypothesis is that epileptic activity arises from excessive positive electrophysiological feedback on excitatory synapses. Loss or damage to inhibitory synapses^3^ and growth or sprouting that results in new synapses^4^^,^^5^ may cause positive feedback. However, many neurobiological processes are implicated in epileptogenesis, and a focus on excitatory/inhibitory imbalance may be limiting.^2^ Inflammation and the immune response, cell growth, plasticity, oxidative stress, and epigenetic reprogramming are all implicated in epileptogenesis and lead to reorganization of circuits via synapse loss, neurite outgrowth, altered synaptic strength, reorganization of the extracellular matrix, damage to the blood-brain barrier, recruitment of inflammatory cells, and gliosis.^6^^,^^7^ Despite this knowledge, the molecular mechanisms and timing of events in epileptogenesis are not clear. Thus, a broad and unbiased view of the protein and signaling landscape is important for further understanding.

Multiple studies using liquid chromatography-tandem mass spectrometry (LC-MS/MS) have been performed in animal models of epilepsy but were either limited in depth or only focused on later stages of epileptogenesis.^8^^,^^9^^,^^10^ Using patient samples, a 2020 study identified greater than 100 differentially abundant proteins in the dentate gyrus^11^ and a recent study found hundreds of changes in the hippocampus and cortex of patients.^12^ Changes to the transcriptome in animal models^13^^,^^14^^,^^15^^,^^16^^,^^17^^,^^18^ or in patient samples^19^^,^^20^ have been examined, however, with limited agreement between studies.^1^^,^^6^ Many existing studies have demonstrated a limited correlation between transcript expression and protein abundance. Therefore, it is important to gain a better understanding of the early phase of epileptogenesis via multiple omics approaches.

Stimulus-dependent phospho-signaling occurs faster than transcriptomic and proteome changes, is dynamic, and can persist to produce a new phosphoproteome status, potentially perpetuating epileptogenic states. Surveying the phosphoproteome also enables identification of key protein kinases involved in signaling through modeling of observed changes to phosphorylation sites that conform to kinase motifs.^21^ Here, we present a large-scale phosphoproteomics and proteomics study of hippocampal tissues from the pilocarpine mouse model of epileptogenesis. In addition to pilocarpine-induced signaling, we also identify the signaling induced by diazepam, a drug used to terminate behavioral seizures.^22^^,^^23^ Data analysis revealed, firstly, that SE induced early phosphorylation of proteins involved in posttranscriptional regulation. Secondly, SE induced dephosphorylation of synaptic proteins. These two major themes extended to related protein abundance changes that supported both growth via translation and synaptic weakening. We found evidence for activation of mitogen-activated protein kinase (MAPK) pathways and mammalian target of rapamycin (MTOR), which are both involved in translation. Also, cyclin dependent-kinase 5 (CDK5) and calcium/calmodulin-dependent protein kinase 2 (CAMK2) substrates, involved in plasticity, were dephosphorylated. The depth of this resource ensures a comprehensive foundation to explore mechanistic leads that might explain protective or detrimental processes occurring in epileptogenesis.

## Results

### Study design

**Figure 1:**
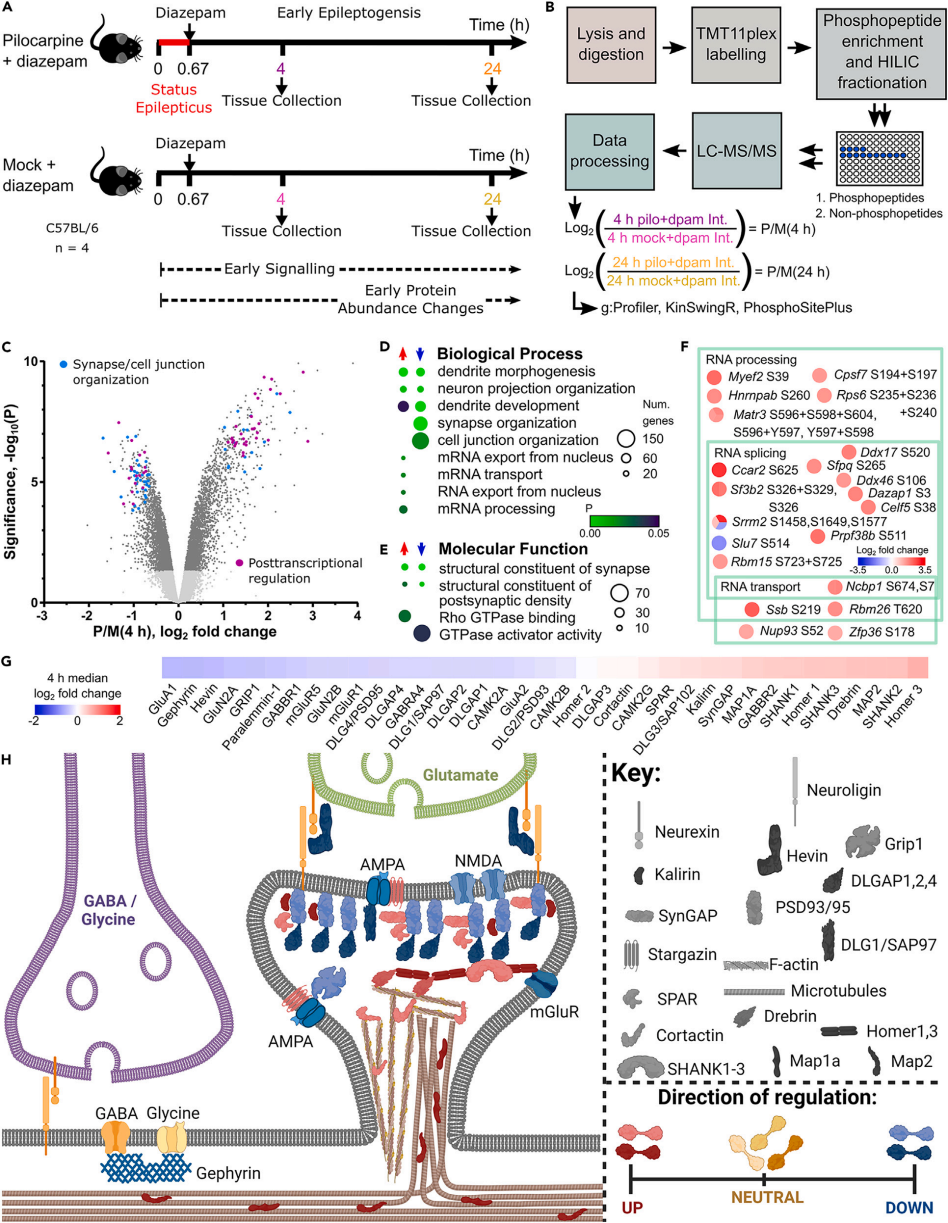
Phosphoproteome changes at 4 h after SE (A) Status epilepticus was induced in mice by pilocarpine or were mock treatment. The hippocampus was dissected at 4 and 24 h. (B) The hippocampus was processed for proteomics. Tryptic peptides were labeled with TMT11plex reagents. Phosphopeptides were enriched and fractionated using the TiSH method prior to LC-MS/MS analysis. The phosphopeptide-depleted sample was also fractionated and analyzed by LC-MS/MS to determine changes to the proteome. The data were log_2_ transformed and specific comparisons were applied in multiple analyses. (C) Volcano plot for the phosphopeptides at 4 h. Light gray dots are values below the threshold for significance. Dots representing the top 50 ranked phosphopeptides from proteins with the terms synapse organization or cell junction organization or related to posttranscriptional regulation are colored. Enriched (D) biological process and (E) molecular function terms. A scale bar for probability is shown. The size of the circle represents the number of genes enriched. (F) Phosphorylation sites of posttranscriptional regulatory proteins ranked in the top 50. The overlapping membership of gene ontology terms is shown. The color of circles represents the log_2_ fold change. (G) Median fold change of selected postsynaptic density (PSD) proteins, transsynaptic proteins, and neurotransmitter receptors (scale bar shown). (H) Schematic representation of the same proteins (descriptively) colored according to the direction of their median phosphorylation change. Created with BioRender.com. See also Figures S1–S3, Tables S1 and S3.

SE was induced in adult mice by application of pilocarpine and terminated 40 min later by injection of diazepam. A second group of mice received a mock treatment, including diazepam (Figure 1A). The hippocampus was dissected at 4 h after treatment, when signaling that persists beyond SE occurs, and at 24 h, when protein abundance changes are well established. However, both time points are still regarded as very early in epileptogenesis. The samples for each time point were lysed, digested, and tagged using the TMT11plex system (Figures 1B and S1A). Phosphopeptides were enriched and fractionated prior to LC-MS/MS analysis. The phosphopeptide-depleted sample was also fractionated and analyzed by LC-MS/MS to determine changes to the proteome (Figure 1B).

Unsupervised principal component analysis showed that replicates for each condition largely clustered into their biological groups (Figures S1B–S1E). TMT reporter ion intensities were log_2_ transformed prior to statistical analysis and the log_2_ fold change was calculated as shown in Figure 1B [abbreviated as P/M(4 h) and P/M(24 h)].

### Deep phosphoproteome analysis of post-SE signaling

We obtained 23,306 unique phosphopeptides with quantitative information from the 4 h TMT11plex set and 22,940 phosphopeptides from the 24 h TMT11plex set. Overall, 28,119 unique phosphopeptides were identified, of which 18,127 phosphopeptides were commonly identified at both 4 and 24 h (Table S1). Phosphopeptides with underlying protein level changes (Table S2) were discarded, leaving 5,375 phosphopeptides with at least one significant change at 4 or 24 h (Figures S2A and S2B). 6,497 phosphopeptides were significantly changed on 2,545 proteins at 4 h and 3,472 phosphopeptides significantly changed on 1,801 proteins at 24 h, which were used for subsequent analyses.

### Gene ontology enrichment and general approach to data analysis

To shed light on how this vast level of phospho-signaling might be influencing cellular functions, we devised an approach to extract relevant information from gene ontology (GO) enrichment. Since SE is a very strong stimulation, we took an approach that deliberately reduced the significant pathways identified by (a) limiting the background to only proteins we could detect by mass spectrometry (Figure S2C) and (b) used the ranked list method within gProfiler,^24^ which weights enrichment toward highly ranked genes. We separated up- and down-regulated phosphorylation, assigned the maximum positive and negative quantitative values for phospho-regulation to each protein, and ranked the proteins using both the quantitative value and significance of maximal change (Table S1).

### Pilocarpine-induced phosphorylation of posttranscriptional regulatory proteins at 4 h

Here, we focus first on phosphorylation changes at 4 h, represented as a volcano plot in Figure 1C and as enriched GO terms in Figures 1D, 1E, S2D, and S2E. A group of biological process terms including “mRNA processing,” “mRNA transport,” and “RNA export from nucleus” and the WikiPathways term “mRNA processing” were associated with increased phosphorylation for Pilo4h (Figures 1D and S2E; Table S3). These terms describe sub-processes of the general process of posttranscriptional regulation and included RNA-binding proteins that target the mRNA of neurotransmitter receptors, encoded by *Sfpq*, *Nova2*, *Celf4*, *Pum1*, *Pum2*, *Eif4enif1*, and *Cpeb4*.^25^ Focusing on the top 50 phosphopeptides with enriched GO terms (Table S1), we found that 27 were from proteins with posttranscriptional regulation terms. While RNA splicing was not enriched at 4 h, many high magnitude phosphorylation increases were on spliceosome or RNA splicing proteins (Figure 1F). There were approximately 240 posttranscriptional regulatory proteins with significant phosphorylation changes. The top 50 ranked phosphopeptide signals involved in posttranscriptional regulation are highlighted in Figure 1C.

### Pilocarpine increased phosphorylation of actin cytoskeleton-linked postsynaptic proteins at 4 h

Multiple biological process terms related to neuron projection or dendrite spine morphogenesis/organization were enriched, whether increased or decreased in phosphorylation (Figure 1D). This bi-directional phosphorylation (simultaneous phosphorylation and dephosphorylation of the same protein) was also observed for related molecular function terms (Figure 1E) and cellular component terms (Figure S2D). To cut through to the underlying meaning of the bi-directional phosphorylation, we determined the median significant log_2_ fold change for individual proteins in the enriched neuronal terms (Table S3), as represented by heatmap in Figure 1G. This approach made clear the proteins that were weighted toward either an increase or decrease in phosphorylation.

A subset of highly ranked postsynaptic proteins with positive median log_2_ fold changes included cortactin, kalirin, microtubule-associated protein 1A (MAP1A), MAP2, drebrin, homer 1/3 and SH3, synaptic Ras GTPase-activating protein (synGAP), and multiple ankyrin repeat domains protein 1-3 (SHANK1-3) (Figure 1G). While cortactin appeared approximately balanced in phosphorylation, closer inspection of the sites indicated that cortactin was converted to a more multi-phosphorylated state, that is, increasing in overall phosphorylation status (Table S1; Figure S3A). Increased phosphorylation of MAP1A at S1898 (Figure S3B) was ranked second of all proteins associated with enriched GO terms (Table S1). Sixty-eight MAP1A sites changed abundance at 4 h and 82% of these were increased (Figure S3B).

Postsynaptic density protein kalirin was one of at least 46 proteins with positive median log_2_ fold changes (Figure 1G) that contributed to the Rho GTPase binding molecular function term enrichment (Figure 1E; Table S3). Many of these proteins promote actin binding, morphogenesis, and/or neurite outgrowth. Thus, pilocarpine-induced phosphorylation occurred on proteins with the potential to alter the cytoskeleton within synapses or sites of neurite outgrowth.

### Proteins linked to neurotransmitter receptors and cell junctions were mainly dephosphorylated at 4 h

A group of biological process terms were significantly associated only with decreased phosphorylation (Figure 1D). The terms synapse organization and cell junction organization encompassed most of these proteins. The top 50 ranked phosphopeptide signals associated with these terms are highlighted in Figure 1C. Examining the higher ranked signals, we found that scaffolding proteins directly or indirectly linked to neurotransmitter receptors or cell junctions were prominent (Figure 1G). For example, disks large-associated protein 1 (DLGAP1) had a negative median log_2_ fold change (Figure 1G).

Postsynaptic density protein 95 (PSD95), disks large family member PSD93, synapse-associated protein 97, and glutamate receptor-interacting protein 1 had a negative median log_2_ fold change (Figure 1G). Most AMPA receptor subunits, N-methyl D-aspartate (NMDA) receptor subunits, and metabotropic glutamate receptors had a negative median log_2_ fold change at 4 h after SE (Table S1; Figure 1G). Ultrastructural data suggest that the postsynaptic density (PSD) is composed of interconnected scaffold layers.^26^ In Figure 1H, we used the median log_2_ fold change of PSD proteins to show that most proteins in the first two scaffold layers beneath the plasma membrane and glutamate receptors had a negative median log_2_ fold change at 4 h, whereas the lower scaffold proteins and MAPs had a positive median log_2_ fold change, indicating differing influence of protein kinases and phosphatases in the scaffold layers.

Gephyrin, the scaffolding protein for inhibitory γ-aminobutyric acid (GABA) and glycine receptors, also had a negative median log_2_ fold change (Figures 1G and 1H). In contrast, GABA receptor subunits had no clear overall phospho-signaling trend at 4 h (Table S1; Figure 1G). The “cell junction organization” set included dephosphorylation of transsynaptic paralemmin 1 (Figures 1G and 1I) and the extracellular matrix protein, hevin (Figures 1G and 1H).

### Known regulatory phosphorylation mechanisms supported increased translation and cell differentiation, but not growth at 4 h

**Figure 2:**
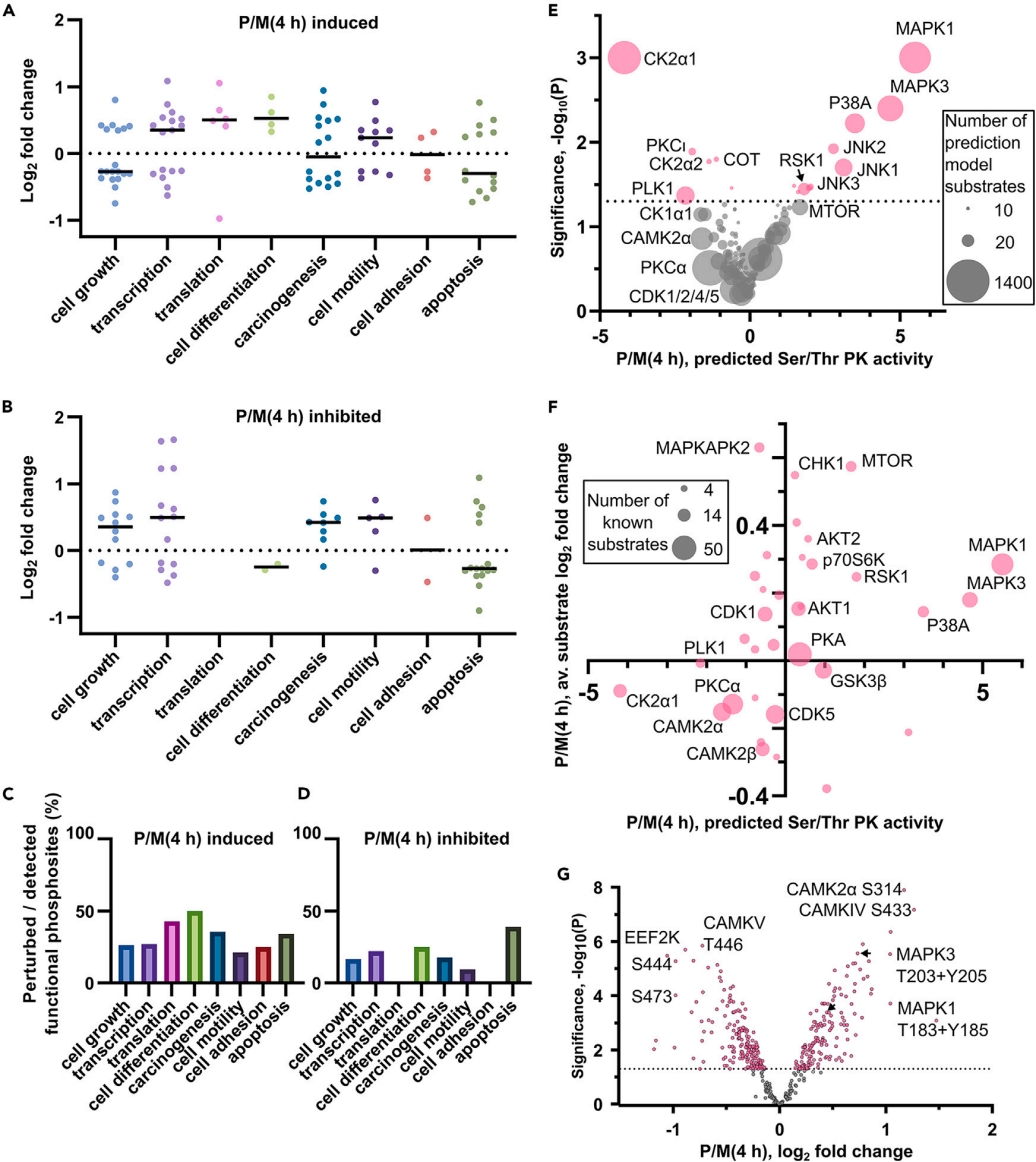
Functional trends and protein kinase prediction for the phosphoproteome analysis at 4 h Log_2_ fold changes are shown for phosphorylation sites with known ability to (A) induce or (B) inhibit various functions at 4 h. The median value is shown for each function as a line. The functional phosphorylation sites detected as perturbed in the phosphoproteome data as a percentage of all detected phosphorylation sites is shown for (C) induced functions and (D) inhibited functions. (E) Volcano plot of KinSwing predicted activity of protein kinases induced by pilocarpine. The size of each dot is scaled to the number of known substrates used to build the protein kinase consensus motif. (F) The average substrate log_2_ fold change for known substrates of protein kinases versus the KinSwing predicted protein kinase activity. (G) Volcano plot of the log_2_ fold changes for phosphopeptides occurring on Ser/Thr protein kinases. See also Figures S4 and S5 and Table S4.

Since the vast majority of phosphorylation sites have no known function, we cannot make generalizations about the effects of increased or decreased phosphorylation. However, we can use those sites with known functions to determine some trends. We identified the processes with evidence for being either induced or inhibited by phosphorylation (according to PhosphoSitePlus^27^ nomenclature). Figures 2A and 2B show the log_2_ fold change for individual phosphorylation sites at 4 h with known functions and the median log_2_ fold change for each function. There was dephosphorylation of sites known to induce cell growth and phosphorylation of sites that inhibit growth, indicating overall inactivation of growth. Translation was induced by phosphorylation (Figure 2A). Greater than 40% of all detected sites that are known to induce translation were perturbed (Figure 2C) and none that inhibit translation were detected (Figure 2B). However, only a few sites regulating translation were present in our dataset (Figure S4) and they are rare in public databases.^28^ There were also few cell differentiation sites detected (Figure S4); however, half of those detected had increased phosphorylated (Figure 2C) and supported induced cell differentiation (Figure 2A). Many sites regulating transcription were detected (Figure S4) but both induction and inhibition were promoted by increased phosphorylation (Figures 2A and 2B). Cell motility and apoptosis also had mixed signals (Figures 2A and 2B). Overall, there were indications that cell growth was prevented while translation and cell differentiation were promoted.

### Protein kinases and phosphatases activated at 4 h implicated MAPK pathways and neuronal plasticity

We used KinSwing, an algorithm we previously developed,^21^ to predict protein kinases upstream of induced signaling. KinSwing uses a network-based algorithm that combines the relationship between quantitative phosphopeptide data and modeled substrate motifs. We may only infer net kinase activity since the contribution from protein phosphatases on phosphorylation site levels is unknown. At 4 h, components of the MAPK signaling pathway, MAPK1, MAPK3, P38A, P38B, c-Jun N-terminal kinase 1 (JNK1), JNK2, JNK3, and ribosomal S6 kinase 1 (RSK1) were predicted from the phosphopeptides to be significantly activated (Figure 2E; Table S4). In contrast, casein kinase 2 α1 (CK2α1) and CK2α2 had decreased inferred activity. Since the detected phosphopeptides included known substrates of protein kinases,^27^ we compared the average significant log_2_ fold change of these known substrates to KinSwing predictions (Figure 2F). The known substrates of MAPK1, MAPK3, P38A, and RSK1 were up-regulated and CK2α1 substrates were down-regulated, validating KinSwing predictions. Increased MTOR activity was also supported by known substrates in Figure 2F despite the activity increase predicted by KinSwing not being statistically significant at the predetermined probability threshold of 0.05 (Figure 2G, p = 0.059).

Direct phosphorylation of protein kinases may cause a change in activity. The known MAPK1 T183 + Y185 and MAPK3 T203 + Y205 activation sites were phosphorylated at 4 h (shown via volcano plot in Figure 2G), confirming the KinSwing prediction (Figure 2E). Multiple phosphosites for CAMK family members were detected (Table S1). The up-regulated phosphorylation of CAMK2α at likely inhibitory site, S314 (Figure 2G), and the autonomous activity site of CAMK2α, T286, was down-regulated at 4 h (Table S1). Even though the prediction for decreased activity of CAMK2α was approaching, but not significant (Figure 2F), validated CAMK2α substrates were, on average, significantly dephosphorylated (Figure 2G). Together, this demonstrated KinSwing predictions were performing well.

The phosphorylation status of several tyrosine protein kinases had relatively large log_2_ fold changes at 4 h (Figure S5A). Both alpha and beta catalytic subunits of the important neuronal plasticity protein phosphatase 2B (PP2B)/calcineurin underwent significant phosphorylation changes at 4 h (Figure S5B). Significant changes were also detected for phosphatase regulatory proteins (Figure S5C) and tyrosine protein phosphatases (Figure S5D). Overall, there was evidence at 4 h for activation of MAPK pathway kinases as well as altered activity of kinases and phosphatases that regulate neuronal plasticity.

### A new phosphoproteome status at 24 h post-SE

**Figure 3:**
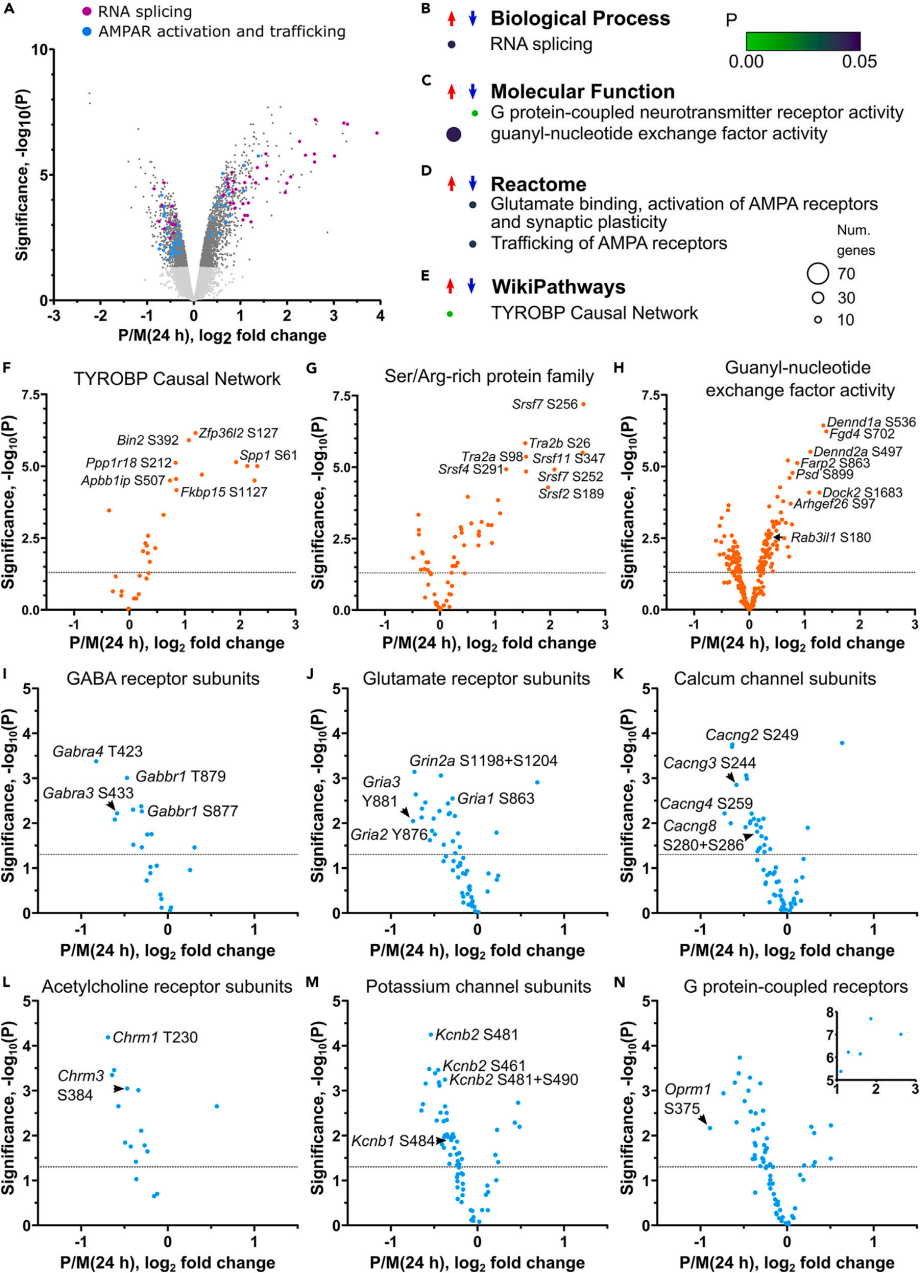
Phosphoproteome changes at 24 h after SE (A) Volcano plot for the significantly regulated phosphopeptides at 24 h. Light gray dots are values below the threshold for significance. Dots representing the top 50 ranked phosphopeptides from proteins with the biological process term RNA splicing and the Reactome terms related to AMPA receptor activation and trafficking are colored. Enriched (B) biological process, (C) molecular function, (D) Reactome, and (E) WikiPathways terms. A scale bar for probability is shown. The size of the circle represents the number of genes enriched. Volcano plots are shown for phosphopeptides with membership of (F) the TYROBP Causal Network, (G) the UniProt curated splicing factor Ser/Arg-rich protein family, (H) guanyl-nucleotide exchange factor activity term, (I) GABA-A receptor activity term, (J) glutamate receptor activity term, (K) voltage-gated calcium channel activity term, (L) acetylcholine receptor activity term, (M) potassium channel activity term, and (N) G protein-coupled receptor activity term. See also Figure S6A, Tables S1 and S3.

**Figure 4:**
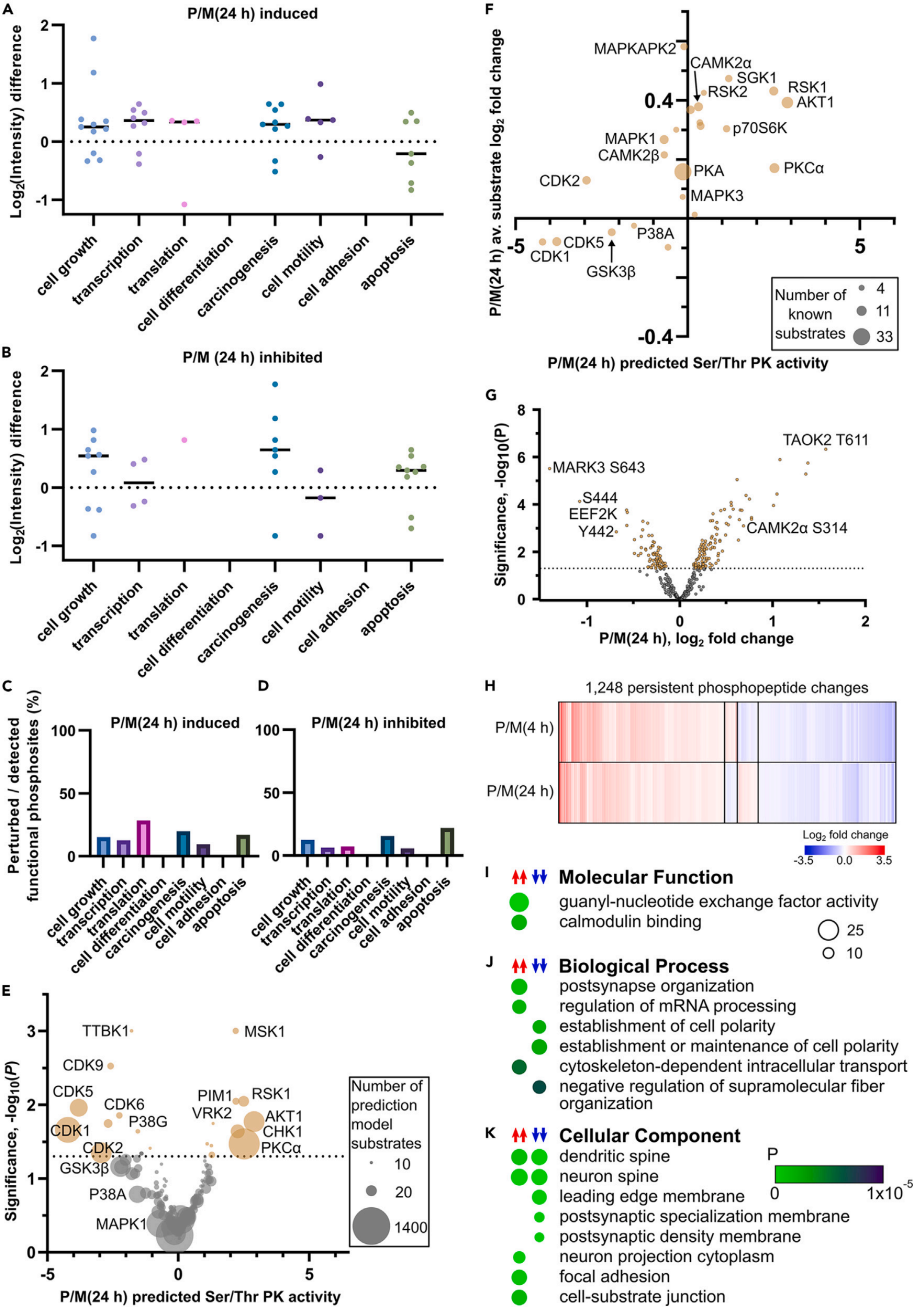
Functional trends and protein kinase prediction for the phosphoproteome analysis at 4 h and persistently regulated phosphorylation Log_2_ fold changes are shown for phosphorylation sites with known ability to (A) induce or (B) inhibit various functions at 24 h. The median value is shown for each function as a line. The functional phosphorylation sites detected as perturbed in the phosphoproteome data as a percentage of all detected phosphorylation sites is shown for (C) induced functions and (D) inhibited functions. (E) Volcano plot of KinSwing predicted activity of protein kinases induced by pilocarpine at 24 h. The size of each dot is scaled to the number of known substrates used to build the protein kinase consensus motif. (F) The average substrate log_2_ fold change for known substrates of protein kinases perturbed by pilocarpine at 24 h versus the KinSwing predicted protein kinase activity. (G) Volcano plot of the log_2_ fold changes for phosphopeptides occurring on Ser/Thr protein kinases at 24 h. (H) Heatmap of phosphopeptides significantly and persistently perturbed at both 4 and 24 h. Enriched (I) molecular function, (J) biological process, and (K) cellular component terms for persistently perturbed phosphoproteins. A scale bar for probability is shown. The size of the circle represents the number of genes enriched. See also Figure S6, Tables S3 and S4.

Only 36% of the phosphopeptides regulated at 4 h were also regulated at 24 h, indicating that there was a new phosphoproteome status at 24 h after SE. A volcano plot of the 3,472 significantly changing phosphopeptides for Pilo24h is shown in Figure 3A. GO enrichment was performed (Figures 3B–3E and S6A; Table S3) using our approach which limits enriched terms to only those with highly ranked phospho-signals. The WikiPathways “TYROBP Causal Network” term was associated with increased phosphorylation at 24 h (Figure 3E) and describes a microglia activation network,^29^ including proteins encoded by the *Spp1*, *Bin2*, *Ppp1r18*, *Samsn1*, *Fkbp15*, and *Apbb1ip* genes (Figure 4F). There were 78 proteins with increased phosphorylation that resulted in the “RNA splicing” term enrichment (Figures 3A and 3B). Looking deeper at the highly ranked RNA splicing factors, we identified the Ser/Arg-rich protein family of splicing factors as a prominent sub-group (Figure 3G). Another group with increased phosphorylation at 24 h were numerous proteins with guanyl-nucleotide exchange factor activity (Figure 3H).

### Inhibitory and excitatory neurotransmitter receptors were dephosphorylated at 24 h

Decreased phosphorylation at 24 h was associated with the molecular function term “G protein-coupled neurotransmitter receptor activity” and the Reactome terms “glutamate binding, activation of AMPA receptors, and synaptic plasticity” and “trafficking of AMPA receptors” (Figures 3C and 3D). These terms included multiple receptor and ion channel subunits (Figures 3A and 3I–3N). Glutamate receptor phosphorylation sites were dephosphorylated at 24 h, for example, the AMPA receptor subunit GluA1 (encoded by *Gria1*) dephosphorylation of S863 (Figure 3J). The transmembrane AMPA receptor regulatory proteins (TARPs) were included in the significantly enriched Reactome terms (Figure 3D) and were dephosphorylated at sites conserved in TARP γ-2, 3, 4, and 8 (Figure 3K). NMDA receptors were not part of enriched Reactome terms but were highly ranked and occurred in most of the enriched cellular component terms (Figure S6A). The 2A subunit (GluN2A, encoded by *Grin2a*) had 13 dephosphorylated sites at 24 h.

Muscarinic acetylcholine receptors are G protein-coupled receptors and followed the trend of decreased phosphorylation at 24 h (Figure 3L), for example, dephosphorylation of S384 of muscarinic acetylcholine receptor M3 (Figure 3L). Since there are many types of G protein-coupled receptors, we asked whether they were all predominantly dephosphorylated at 24 h in our data. We found that most receptor phosphorylation site changes involved decreased phosphorylation (Figure 3N). An example is the μ-opioid receptor (Figure 3N).

### Pilocarpine induced dephosphorylation of potassium channels at 24 h

Potassium channels were highly ranked in five of the cellular component terms associated with decreased phosphorylation (Figure S6A). The delayed rectifiers Kv2.1 and Kv2.2 (encoded by *Kcnb1* and *Kcnb2*) had multiple down-regulated phosphorylation sites (Figure 3M) and two sites were decreased for epilepsy-associated Kv2.1 (encoded by *Kcnb1*) (Figure 3M).

### Functional phosphorylation sites support cell growth, cell motility, and inhibition of apoptosis at 24 h

Using curated information of known functional phosphorylation sites, it became clear that cell motility was likely induced by phospho-regulation at 24 h after SE, without significant inhibition (Figures 4A–4D). Cell growth was induced, as was the associated process of carcinogenesis (Figure 4A), although both were simultaneously inhibited by a lesser number of functional phosphorylation sites (Figure 4B). Phospho-regulated inhibition of apoptosis was also clearly supported at 24 h (Figure 4B). Functional phosphorylation sites for apoptosis were well represented in the data (Figures 4C and 4D), although the overall level of perturbed functional phosphorylation sites was decreased at 24 h relative to those at 4 h (Figures 2C and 2D).

### Protein kinase and phosphatase activity at 24 h implicates signaling involved with cell growth and chronic synaptic activity

At 24 h, multiple CDKs had decreased inferred activity, using KinSwing (Figure 4E; Table S4). CDK1 and CDK5 substrates had the most evidence for decreased phosphorylation using the average fold change for known substrates (Figure 4F). Mitogen and stress response kinase 1 (RSK1) and protein kinase B (AKT1) were predicted to be up-regulated (Figure 4E) and had known substrates with increased phosphorylation (Figure 4F), implicating signaling pathways associated with the stress response and cell growth.

Direct phosphorylation of protein kinases at 24 h did not implicate many phosphorylation sites with known function. However, we observed CAMK2α inhibitory S314 phosphorylation at 24 h (Figure 4G), which was diminished relative to the phosphorylation level at 4 h (Figure 2H), and phosphorylation of ephrin type-A receptor 4 phosphorylation at Y779 (Figure S6B). There were fewer changes detected for phospho-regulated phosphatases at 24 h (Figures S6C–S6E) than at 4 h.

### Persistent signaling from 4 to 24 h after SE supports main findings

There were 1,248 phosphopeptides significantly changed at both 4 and 24 h post-SE (Figure 4H), which was too small to obtain sufficient enriched GO terms with the procedure used for the single time points. After relaxing the background to include the entire mouse proteome, we identified enriched terms (Figures 4I–4K; Table S3) consistent with our trends. The persistently phospho-regulated proteins were mainly those with guanyl-nucleotide exchange factor activity, involved in RNA processing or located at the postsynapse.

### Diazepam induced phosphorylation of cytoskeletal and plasticity proteins while GABA receptor subunits were dephosphorylated

**Figure 5:**
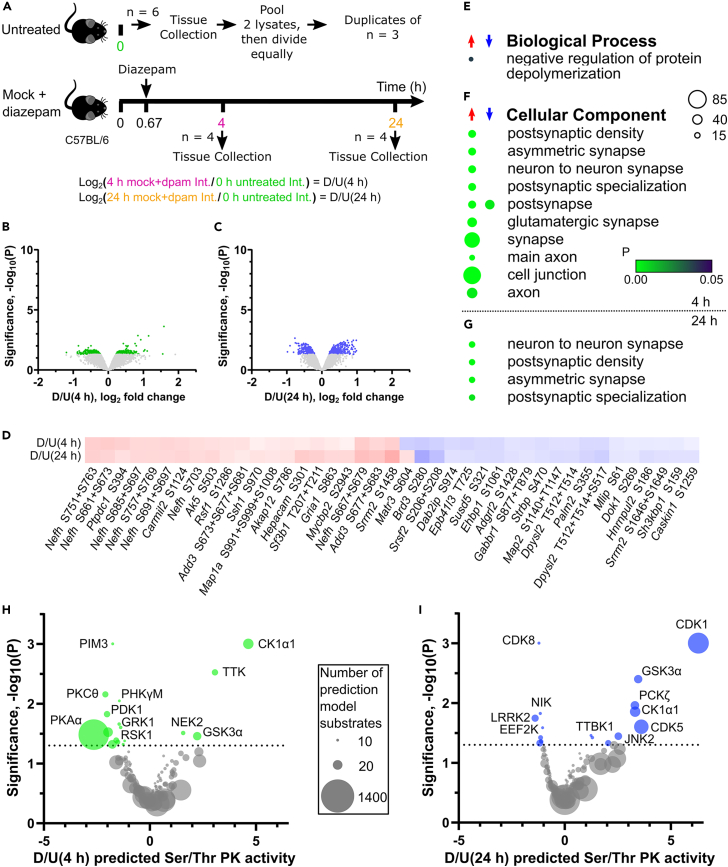
Phosphoproteome changes induced by diazepam at 4 and 24 h (A) Mice receiving a mock injection followed by diazepam injection at 40 min were compared to untreated mice. Volcano plots for the diazepam-induced phosphorylation level changes at (B) 4 h and (C) 24 h. Light gray dots are below the threshold for significance. Enriched (E) biological process terms at 4 h, (F) cellular component terms at 4 h, and (G) cellular component terms at 24 h after mock injection. A scale bar for probability is shown. The size of the circle represents the number of genes enriched. (D) Heatmap of persistently perturbed phosphorylation sites after diazepam. Volcano plots of KinSwing predicted activity of protein kinases induced by pilocarpine at (H) 4 h and (I) 24 h. The size of each dot is scaled to the number of known substrates used to build the protein kinase consensus motif. See also Tables S1, S3, and S4.

We used a group of mice that received no treatments or injections (“untreated”) as a control to investigate the specific effects of diazepam injection (Figure 5A), a first-line treatment for SE. We identified 503 and 541 significant phosphorylation-level changes, respectively, at 4 and 24 h post-mock injection that were induced by diazepam (Figures 5B and 5C; Table S1). There were 754 phosphopeptide changes significant at one or more time points. Forty-one changes were significant at both time points (Figure 5D). The term “negative regulation of protein depolymerization” was enriched and associated with increased phosphorylation at 4 h after diazepam treatment (Figures 5E and 5F). This term included the cytoskeletal proteins MAP1A, MAP1B, MAP2, β-adducin, and spectrin-α non-erythrocytic 1 (Table S3). Diazepam altered the phosphorylation of its target GABA receptor at 4 h, mainly with dephosphorylation, which contributed to enrichment of the “postsynapse” cellular component term (Figure 5D). However, most synaptic proteins had up-regulated phosphorylation (Figure 5F), including AMPA and NMDA receptor subunits, such as S863 on GluA1 (Figure 5D). Increased phosphorylation was also associated with synaptic terms at 24 h (Figure 5G). Neurofilament heavy chain had increased phosphorylation at multiple sites, which was significant at both 4 and 24 h after diazepam (Figure 5D).

When we used KinSwing to investigate inferred protein kinase activity, we found that CK1α1 had the greatest inferred activity at 4 h (Figure 5H). CDK5 increased inferred activity at 24 h after diazepam (Figure 5I). Further, a known substrate of CDK5, β-2-syntrophin S75,^30^ increased phosphorylation at 24 h after diazepam, again supporting the prediction that CDK5 is affected by diazepam treatment.

### Proteome changes at 4 h after SE indicated MAPK pathway stimulated immediate-early gene expression

**Figure 6:**
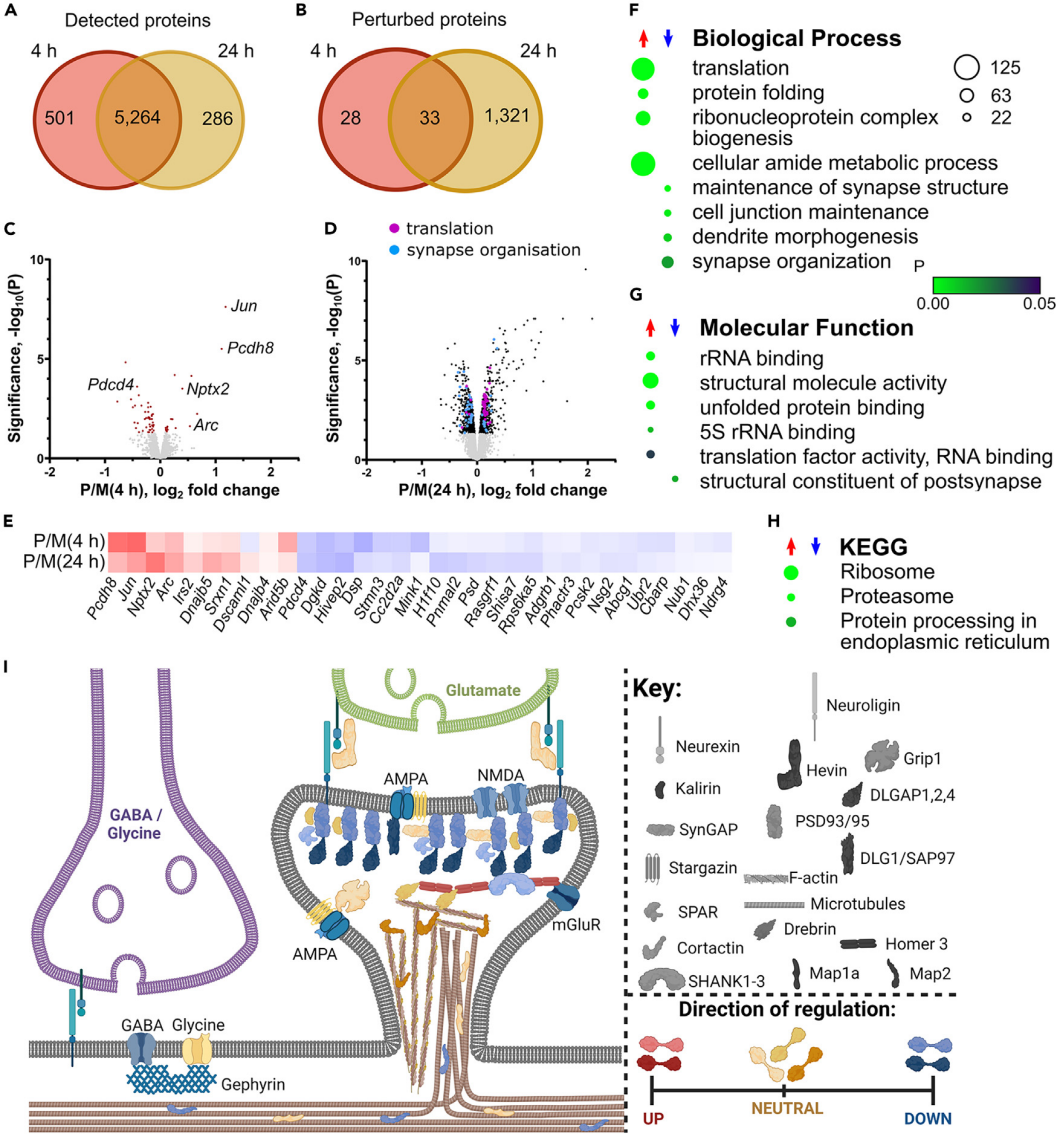
Proteome changes at 4 and 24 h after SE Venn diagram of (A) proteins detected and (B) perturbed at each time point, resolved to non-redundant gene names. Volcano plot of protein changes at (C) 4 h and (D) 24 h after SE. Light gray dots are below the threshold for significance. The top 50 ranked proteins with the biological process terms translation and synapse organization are shown as colored dots. (E) Protein changes persistently detected at 4 and 24 h after SE. Enriched (F) biological process, (G) molecular function, and (H) KEGG terms at 24 h. A scale bar for probability is shown. The size of the circle represents the number of genes enriched. (I) Schematic representation of the postsynaptic compartment with (descriptive) coloring according to direction of protein regulation. Created with BioRender.com. See also Figures S7 and S8, Tables S2 and S5.

The proteomic screen detected 6,277 protein groups (Table S2). Protein groups are made up of one or more proteins that have evidence for existence from the detected peptides. The 6,277 protein groups mapped to 6,051 non-redundant gene names. When phosphopeptide-based protein identifications were allowed in the count (requiring at least two phosphopeptides), the number of non-redundant genes increased to 7,553 (Figure S2C). After discarding protein mapping to the same gene, there were 5,765 and 5,550 proteins in the 4 and 24 h TMT11plexes, respectively, which intersected on 5,264 proteins (Figure 6A; Table S2). 1,328 proteins were significantly perturbed at one or more time points after SE (Figure 6B).

Sixty-one proteins had significant level changes 4 h post-pilocarpine treatment and most of these had decreased abundance (Figure 5C). The regulated proteins were analyzed for GO enrichment after separation into sets of up- and down-regulated proteins. Only the WikiPathways term “p38 MAPK signaling pathway” was significantly enriched at 4 h (Table S5). The levels of two immediate-early genes, Jun and Arc (Figure 5C), were significantly up-regulated at 4 h with Jun being the most significantly up-regulated protein, which is a hallmark of early SE (Table S6). Further proteins linked to neuronal activity also had increased abundance, protocadherin 8 (encoded by *Pcdh8*) and neuronal pentraxin 2 (encoded by *Nptx2*) (Figure 6C). Jun, Arc, protocadherin 8, and neuronal pentraxin 2 were also among 10 persistently up-regulated proteins (Figure 6E). There were more persistently down-regulated proteins at 4 h, but our analyses did not associate any specific GO terms.

### The proteosome, ribosome proteins, and translation factors had increased expression at 24 h

Approximately 25% of the detected proteome had significantly changed abundance 24 h after pilocarpine injection (Figures 6B and 6D). Enriched GO terms indicated that the main proteins with increased abundance at 24 h were involved in translation (Figures 6F–6H and S7A–S7C; Table S5). More than 70 ribosomal proteins increased abundance, covering more than half of the small and large ribosomal subunits. At least 20 eukaryotic initiation factor subunits increased abundance (Table S5).

Associated with increased abundance were “protein folding” and “unfolded protein binding” terms (Figures 6F and 6G), which further indicated an increased use of protein synthesis mechanisms. In contrast, there was increased abundance of 20 components of the proteosome complex (Table S5) resulting in enrichment of the “Proteasome” Kyoto Encyclopedia of Genes and Genomes term (Figure 6H) and “proteosome degradation” WikiPathways term (Figure S7C).

### Pilocarpine induced a decrease in abundance of synaptic proteins at 24 h

Synaptic-related terms were associated with reduced protein abundance at 24 h (Figures 6F and 6G), including glutamate receptor subunits GluN2B, GluA4, and metabotropic glutamate receptor 5. Postsynaptic density proteins PSD93, PSD95, DLG1/SAP97, DLGAP1/2/4, SH3, SHANKs 1–3, and homer 2 were decreased. Also reduced were presynaptic proteins, transsynaptic proteins, and cytoskeletal proteins. Overall, decreased postsynaptic density protein levels were mainly observed for the upper and middle layers of the PSD scaffold (Figure 6I). Proteins specific to GABAergic neurons, including gephyrin, neuroligin 2, five GABA receptor subunits, glutamate decarboxylase 1 and 2, and the potassium-chloride cotransporter were also decreased at 24 h.

### Diazepam induced a decrease in mitochondrial respiratory chain proteins

Diazepam injection had no detectable effect on the proteome at 4 h compared to untreated controls. Approximately 3% of the detected proteome was affected 24 h after diazepam injection (Figures S8A and S8B). The main class of proteins producing significantly enriched terms were mitochondrial respiratory chain proteins (Figures S8C–S8G; Table S5), which were decreased.

### Independent experimental validation confirms the main findings are reproducible

**Figure 7:**
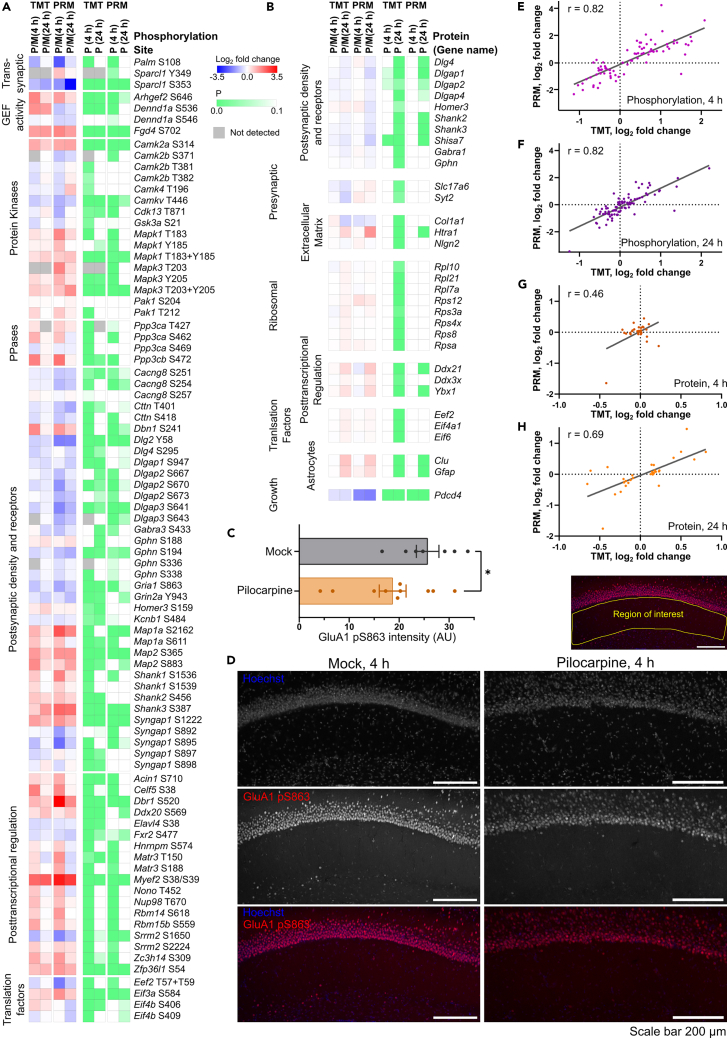
Independent analysis of targets by parallel reaction monitoring Heatmap of (A) phosphorylation site and (B) protein changes observed by PRM at 4 and 24 h alongside the findings by TMT and the probabilities for each. Scale bars are shown. (C) Immunolabeling of hippocampal slices from mock or pilocarpine-treated mice. The slices were stained with Hoechst to mark nuclei or anti-GluA1 pS863. A region of CA1 neurites was defined (example in upper right panel). The intensity of staining was quantified and compared between the control and pilocarpine-treated samples (∗, p = 0.032, one-sided t test for a decrease). Data are represented as mean ± SEM. (D) Representative images. The PRM measurements for phosphorylation sites at (E) 4 h and (F) 24 h as well as the PRM measurements for protein at (G) 4 h and (H) 24 h plotted against the corresponding TMT measurements are shown. The Pearson correlation coefficient and linear regression line for the measurements is shown on each graph. Scale bars represents 200 μm. See also Table S7.

We produced a new set of experimental samples using the same pilocarpine mouse model and analyzed those samples using the targeted MS method of parallel reaction monitoring (PRM).^31^ Over 80 phosphorylation sites (Figure 7A) and 32 proteins (Figure 7B) were targeted by PRM (Table S7). These targets represented different classes of proteins found to be enriched in the GO analysis derived from our primary TMT data. These PRM data supported our TMT data. For example, posttranscriptional regulators had mainly increased phosphorylation and protein kinases such as MAPK1/3 increased in phosphorylation at activating sites. Furthermore, PSD proteins were segregated into groups that had increased and decreased phosphorylation. The GluA1 S863 phosphorylation site was decreased in both TMT and PRM experiments at 4 h. Validation of the dephosphorylation by immunolabeling of hippocampal slices also indicated a reduction in the CA1 region at 4 h, (Figures 7C and 7D, p = 0.032). Notably, some phosphorylation sites did not reach significance by PRM and a minority showed changes in phosphorylation that were opposite to the TMT measurement. However, the overall finding was that both the 4 and 24 h phosphorylation site level measurements by PRM were highly correlated to the TMT data (Figures 7E and 7F).

Protein changes were also supported by PRM. PSD proteins that had mostly decreased abundance at 24 h by TMT were also decreased in abundance by PRM (Figure 7B). Astrocytic proteins, glial fibrillary acidic protein (encoded by *Gfap*), and clusterin (encoded by *Clu*) had increased abundance. Two presynaptic protein targets (encoded by *Syt2* and *Slc17a6*) gave the opposite result (Figure 7B). The ribosomal proteins, that had increased abundance using TMT, were not significantly increased via PRM. In general, PRM measurements were greater in magnitude (Figures 7E–7H, y axis) than the TMT measurements but less significant (Figures 7A and 7B). This is likely due to the different methods and the different scale of data, leading to different processing and statistical treatment. The log_2_ fold changes for protein at 4 h were expected to be small and they were (Figure 7G). The TMT and PRM protein data at 24 h were highly correlated (Figure 7H).

## Discussion

SE triggers a large number of changes in the structural organization and cellular composition of the hippocampus as well as in the electrophysiological properties of the neuronal network which ultimately result in the manifestation of spontaneous self-recurring chronic seizures. Since neuronal activity impacts the cellular properties of brain cells, in particular of neurons, the pathological strong neuronal activity induced by SE is likely to trigger cellular signaling cascades that underlie epileptogenesis. However, these signaling cascades have not been detailed at the protein level, until this work. Here we find that early phospho-signaling is directed toward controlling posttranscriptional regulation and postsynaptic functions. Also, the earliest protein changes are supportive of translation but result in decreased postsynaptic proteins. Our initial analysis using TMT was validated by a targeted analysis of representative proteins and phosphorylation sites using PRM on independent samples. Although not always reaching statistical significance, the PRM data were highly correlated to the TMT data, supporting the reproducibility of our findings.

### Reduced phosphorylation and protein expression of proteins involved in neuronal plasticity after SE

It is well established that neuronal networks aim to maintain their firing rate within a physiological target range by homeostatic regulation of synaptic strength.^32^ Neurons experience strong pathophysiological activity during SE. The limited electrophysiological studies in rats^33^^,^^34^ and mice^35^ suggest that long-term potentiation (LTP) is impaired as an early compensatory mechanism opposing strong activity. Our data strongly support a homeostatic synaptic response.

The interplay of CAMK2α and PP2B controls the phosphorylation status of postsynaptic proteins and neurotransmitter receptors, thereby regulating synaptic strength during plasticity.^36^ Our results show that SE results in changes in the activity of these two proteins. Changes in the phosphorylation status of the CAMK2α autophosphorylation sites indicated that the kinase was deactivated at 4 and 24 h after SE and, fittingly, most predicted and known CAMK2α substrates were dephosphorylated at 4 h after SE. However, there were also examples indicative of prior hyperphosphorylation by CAMK2α, such as the increased phosphorylation of homers (homer1 S117 and homer3 S159) which are known to cause the dissolution of homer’s interaction with metabotropic glutamate receptor 5 and circuit hyperexcitability.^37^ In accordance with an overall decrease of CAMK2α activity, our data suggest that calcium-dependent phosphatases are active at 4 h after SE. Both low and high frequency action potentials activate PP2B, which has a higher affinity for calmodulin than CAMK2.^38^ Supportive of this, we found that phosphorylation sites on PP2B which are known to inhibit PP2B activity were dephosphorylated in our model.^39^^,^^40^ Furthermore, we observed dephosphorylation of known PP2B substrates. Dephosphorylation of S863 on GluA1, which was validated by PRM and immunolabeling, is known to be mediated by PP2B and to cause receptor internalization and long-term depression (LTD).^41^ We observed TARP dephosphorylation, which is downstream of PP2B and is associated with AMPA receptor trafficking and LTD.^42^ Presynaptically, synaptic strength can be modulated by the interplay of CDK5 and PP2B.^43^ Postsynaptic CDK5 function is less understood, but has some important substrates. For example, neurotrophic receptor tyrosine kinase 2 was dephosphorylated at a CDK5 substrate site which is required for activity-dependent structural plasticity associated with LTP.^44^ There was evidence of synaptic downscaling at 24 h, which included dephosphorylation of EEF2K substrates,^45^ dephosphorylation of DLGAP1 S499,^46^^,^^47^ phosphorylation of synGAP S842,^48^ and phosphorylation of ephrin type A receptor 4 Y779.^49^ The RNA splicing protein Nova2 was phosphorylated at T27 at 4 h after SE and is involved in the regulation of LTP and homeostatic plasticity via alternative splicing of synaptic proteins.^50^^,^^51^ Taken together, our data suggest that an early response to SE is the activation of predominantly phosphatase-mediated regulation of postsynaptic receptor complexes, and other proteins broadly involved in neuronal plasticity, to alter synaptic strength, which suggests an early neuroprotective homeostatic response. Subsequent reduced abundance of the postsynaptic receptor complex is suggestive of weakened synaptic strength, synapse loss,^52^ or neuronal cell death.^53^

### SE triggers changes in the phosphorylation status of proteins associated with synaptic and neuronal reorganization

Pilocarpine-induced SE has been shown to result in dendritic spine loss.^54^ Changes in the phosphorylation status of PSD scaffolding proteins and cytoskeletal proteins present in dendritic spines within our data might contribute to dendritic structural plasticity. For example, all SHANKs had increased phosphorylation at 4 h after SE. SHANK3 activity has been shown to regulate spine number and size.^55^ Also, the phosphorylation status of multiple proteins associated with the cytoskeleton, for example MAP2 and drebrin, was mainly increased early after SE. These postsynaptic scaffold components exist in a layer furthest from the membrane, which is a route of access to the PSD for CAMK2^26^ and it is striking that they have similar increased phosphorylation.

In addition, cell junction proteins such as paralemmin-1 and hevin were heavily dephosphorylated; however, the functions of the dephosphorylated sites are unknown. Hevin is implicated in epilepsy since it accumulates at excitatory synapses at 24 h in a rat pilocarpine seizure model^56^ and phospho-regulation of paralemmin may influence its role in membrane remodeling and synapse formation.^57^ Dysregulated cell junction proteins such as paralemmin-1 and hevin could contribute to synapse destabilization.

### Evidence for MAPK pathway signaling potentially leading to posttranscriptional regulation and cell growth

We found evidence of MAPK pathway signaling, including the extracellular signal-regulated kinase, P38, RSK, and JNK families, at 4 h after SE. MAPK pathways are known to target transcription activators in response to stress, growth factors, and neuronal activity.^58^ MAPKs mainly activate transcription, but also the MTOR pathway, which is a known activator of translation.^59^ There is growing evidence that MAPKs and MTOR are implicated in epilepsy by phosphorylating and regulating RNA-binding proteins,^25^^,^^59^ for which our unbiased screen gives further support. MTOR inhibition has been shown to have favorable disease-modifying effects.^59^^,^^60^^,^^61^

Strikingly, posttranscriptional regulatory proteins were the major targets of phosphorylation, at both 4 and 24 h after SE. The brain uses alternative splicing more than any other organ and has an expanded set of posttranscriptional regulatory mechanisms to match.^25^^,^^62^ However, there have been few mechanistic studies of posttranscriptional regulatory proteins.^28^ Our study revealed that posttranscriptional regulatory proteins that span the life cycle of an RNA molecule, including splicing, export, transport, translation, and degradation,^25^ were major substrates of early signaling.

The RNA-binding proteins encoded by *Celf4* and *Sfpq* were prominently phospho-regulated. *Sfpq* is important for axon survival and *Celf4* regulates neurotransmitter receptor abundance.^25^ Since RNA-binding proteins have cell-type-specific functions that control neurotransmitter receptor abundance, their perturbation is likely to affect excitatory/inhibitory balance. The RNA-binding protein, pumilio 2, was phospho-regulated in our data and has been implicated in epilepsy,^63^ but so far there are few such examples of RNA-binding proteins related to epilepsy. It is not known if phosphorylation of a broad group of posttranscriptional regulatory proteins leads to mainly increased or decreased translation and which cell types are most affected. The major factors will need to be investigated further to reveal their role in epileptogenesis.

At 24 h, ribosomal proteins, translation initiation factors, and proteins regulating folding had increased expression. Ribosome proteins can be locally synthesized and selectively bind to 3′-untranslated regions creating specialized ribosomes for regulating the specificity and efficiency of local protein synthesis.^25^ The detection of approximately 20 proteosome components with increased abundance at 24 h post-SE suggests that protein degradation was highly activated at the same time that protein synthesis was promoted. The proteosome is required for both apoptosis, to remove unwanted protein, and as a protein quality control mechanism in cell growth.^64^ It is notable that dysregulated proteostasis is a common feature of neurological diseases. For example, in models of hyperexcitability in fragile X syndrome.^65^ The signals for both ribosome and proteasome protein increases were relatively weak and will require further investigation.

There were multiple additional indicators of cell growth. Many Rho GTPase proteins had up-regulated phosphorylation at 4 h and guanyl-nucleotide exchange factors had persistently increased phosphorylation at both 4 and 24 h. Many of these proteins are known to mediate cytoskeletal functions which lead to morphogenesis or cell growth.^66^ Also, there was increased phosphorylation of sites known to promote cell migration. The mechanisms for cell growth and migration are highly relevant to understanding epileptogenesis since growth or sprouting that might be necessary for repair may also lead to more excitatory synapses, causing positive feedback and more frequent seizures.^4^^,^^5^ Protein kinases associated with growth signaling, such as MTOR and AKT1, had positive indicators for increased activity. MTOR is also known to contribute to an immune response. There was only minor evidence of microglia activation, beginning with a few relevant protein changes at 24 h. Thus, signals indicative of inflammation were present but relatively weak compared to indicators of posttranscriptional regulation and growth.

### Diazepam induced synaptic plasticity mechanisms and decreased expression of mitochondrial respiratory chain proteins

In the pilocarpine animal model of epileptogenesis, diazepam is used to allow mice to recover from SE due to activation of GABA receptors that inhibit excitatory synapse depolarization. Humans routinely use diazepam or homologous drugs to induce sleep. However, to our knowledge, there is limited understanding about the mechanistic effect of diazepam in the 24 h after administration. Surprisingly, we found that diazepam application on its own caused significant alterations in the hippocampal phosphoproteome. For example, proteins involved in GABAergic signaling were phospho-regulated, GluA1 was phosphorylated at LTP-associated S863^41^^,^^67^ and there were phosphorylation changes on methyl-CpG-binding protein 2, a protein which regulates synaptic scaling.^68^ The direction of phosphorylation change on GluA1, in particular, supports previous findings at the functional level that diazepam can induce synaptic upscaling functionally similar to that induced by tetrodotoxin.^69^^,^^70^ Reduced abundance of proteins involved in the mitochondrial respiratory chain was the major protein change following diazepam at 24 h. Since diazepam suppresses brain activity, likely, there was reduced energy metabolism. Our data call for future studies investigating whether there are even longer term effects (>24 h) following a single diazepam application and whether they might be related to the development of addictions.

### Limitations of the study

We acknowledge that a major limitation of this work is that measurements from whole hippocampus cannot be used to resolve changes that are specific for certain cell types or subcellular compartments and proteins can have a different response to stimuli in different locations.

## STAR★Methods

### Key resources table

**Table:** 

REAGENT or RESOURCE	SOURCE	IDENTIFIER
**Antibodies**		

Anti-GluA1 pS863 (rabbit)	Invitrogen	PA5-104896
Goat anti-Rabbit IgG (H+L) Cross-Adsorbed Secondary Antibody, Alexa Fluor 568	Invitrogen	A-11011

**Chemicals, peptides, and recombinant proteins**		

10% w/v sodium dodecyl sulfate solution	BIO-RAD	1610416
Complete, EDTA-free Protease Inhibitor Cocktail	MERCK/Roche	04693132001
PhosSTOP	MERCK/Roche	PHOSS-RO
Phenylmethanesulfonyl fluoride	MERCK/Sigma	P7626-5G
Tris(2-carboxyethyl)phosphine hydrochloride solution	MERCK/Sigma	646547-10X1ML
Iodoacetamide	MERCK/Sigma	MERCK
Lysyl Endopeptidase (Lys-C) for biochemistry	Novachem/FUJIFILM Wako	129-02541
TrypZean bovine	MERCK/Sigma	T3568-10MG
TMT10plex reagents	Thermo Fisher Scientific	90111
TMT11-131C Label Reagent	Thermo Fisher Scientific	A37724
hydroxyethyl]-1-piperazineethanesulfonic acid (HEPES)	MERCK/Sigma	H3375-100G
Urea	Astral Scientific	BIOUB0148 - 500G

**Deposited data**		

TMT phosphoproteome and proteome	This paper	PRIDE: PXD038241
PRM to quantify phosphorylation sites and proteins	This paper	PRIDE: PXD047870

**Experimental models: Organisms/strains**		

Mouse: C57Bl6/N mice	Charles River	N/A

**Software and algorithms**		

MaxQuant 1.6.7.0	Tyanova et al.^71^	https://www.maxquant.org/
Skyline 23.1.0.268	MacLean et al.^72^	https://skyline.ms/wiki/home/software/Skyline/page.view?name=default
limma 3.36.5	Ritchie et al.^73^	http://bioconductor.org/packages/release/bioc/html/limma.html
Morpheus	N/A	https://software.broadinstitute.org/morpheus/
Mascot 2.7.0	Perkins et al.^74^	http://www.matrixscience.com
KinSwingR	Engholm-Keller et al.^21^	http://bioconductor.org/packages/KinSwingR/
gProfiler	Raudvere et al.^24^	https://biit.cs.ut.ee/gprofiler/gost
ImageJ 1.54h	National Institutes of Health	https://imagej.net/ij/download.html
Revigo	Supek et al.^75^	http://revigo.irb.hr/
GraphPad Prism 9.0.2	GraphPad Software	N/A
Excel 2016	Microsoft	N/A

### Resource availability

#### Lead contact

Further information and requests for resources and reagents should be directed to and will be fulfilled by the lead contact, Mark Graham (mgraham@cmri.org.au).

#### Materials availability

This study did not generate new unique reagents.

#### Data and code availability

Data reported in this paper will be shared by the lead contact upon request. All mass spectrometry data have been deposited at PRIDE and are publicly available as of the date of publication. Accession numbers are listed in the key resources table. This paper does not report original code. Any additional information required to reanalyze the data reported in this paper is available from the lead contact upon request.

### Experimental model and study participant details

#### Animals

All animal procedures were planned and performed to minimize pain and suffering and to reduce the number of used animals in accordance with the guidelines of the University Hospital Bonn, Animal-Care-Committee as well as the guidelines approved by the European Directive (2010/63/EU) on the protection of animals used for experimental purposes and ARRIVE guidelines. All mice were housed in a humidity (55 ± 10 %) and temperature (22 ± 2°C) controlled environment under a 12-h light–dark-cycle (light cycle 7 am to 7 pm) with water and food *ad libitum* and nesting material (Nestlets, Ancare, USA). All mice were male C57Bl6/N strain (Charles River; ∼60 days old, weight ≥ 20 g) and were allowed to adapt to the animal facility at least for seven days before any treatment.

### Method details

#### Induction of chronic epilepsy

The mice were injected subcutaneously with 335 mg/kg pilocarpine hydrochloride (Sigma) to induce status epilepticus, 20 minutes after pre-treatment with subcutaneous injection of 1 mg/kg scopolamine methyl nitrate (Sigma).^22^^,^^23^ Animals received an injection of diazepam (4 mg/kg, s.c.; Ratiopharm) forty minutes after SE onset. Control animals were treated identically but received saline instead of pilocarpine (mock treated). Behavioural SE was clearly identified using a modified seizure scheme: sustained convulsions with postural loss designated as SE.^22^^,^^23^ Among pilocarpine injected animals, only those that developed SE (SE-experienced) were further used for analysis.

#### Preparation of hippocampi lysates

4 h and 24 h post induction of SE hippocampi from both hemispheres were removed and immediately frozen on dry ice. Tissue was homogenized in 500 μl PBS (45 sec), before 125 μl lysis buffer was added consisting of 2% sodium dodecyl sulphate, 50 mM trisaminomethane/HCl pH 7.4, 2 mM ethylene glycol-bis(β-aminoethyl ether)-N,N,N0, N0-tetraacetic acid, 2 mM ethylenediaminetetraacetic acid, Complete Protease Inhibitor Cocktail (Roche), 2 mM phenylmethylsulfonyl (Sigma), 5 mM NaF (Sigma), 2 mM beta-glycerophosphate (Sigma), Phosphatase Inhibitor Cocktail 2 (1:1000) and PhosSTOP (Roche). Lysates were incubated at 85°C for 10 min and subsequently sonicated for 3 x 10 s. Samples were frozen on dry ice before being lyophilized for 60 h.

#### Sample processing for proteomics analysis

The freeze-dried samples were resuspended in a 100 μL solution of 10 mM tris(2-carboxyethyl) phosphine, reduced for 10 minutes at 85°C and subsequently alkylated in 25 mM iodoacetamide at 22°C in the dark. Proteins were precipitated from the samples using chloroform-methanol extraction^76^ and air dried. The precipitates were dissolved in 20 μL solution containing 7.8 M urea, 50 mM triethylammonium bicarbonate and 5 μg of Lys-C (FUJIFILM Wako Pure Chemical Corporation) for an 8 h digestion at 25°C. The samples were diluted with a solution containing 170 μL 100 mM 4-[2-hydroxyethyl]-1-piperazineethanesulfonic acid (HEPES) (pH 8) and 5 μg TrypZean trypsin (Sigma Life Sciences) for subsequent digestion at 37°C for 4 h. The trypsin digestion was twice repeated with an additional 5 μg for 4 h at 37°C. The peptide concentration was estimated by absorption at 280 nm (Implen Nanophotometer, Labgear).

#### TMT labelling and phosphopeptide enrichment

Approximately 400 μg of peptide was aliquoted into new tubes of equal concentration, using 100 mM HEPES (pH 8) to adjust the volume. TMT11plex reagent sets (Thermo Fisher Scientific) were dissolved in anhydrous acetonitrile and applied to each of the twenty-two samples in a 1 h and 20 min incubation at 25°C, using one reagent vial per 200 μg. To ensure completeness of labelling 0.6 μL from each sample was tested and were found to be > 98% labelled. Excess TMT reagent was quenched with 16 μL of a 5% hydroxylamine solution for 15 minutes at 25°C. The 4 h and 24 h treatment samples were pooled separately according to their respective timepoints. The six control untreated replicates were then pooled together and thoroughly mixed before splitting the combined sample in half, to mix with the two timepoint treatments. The 4 h TMT sample set consisted of: control untreated bio-replicate 1, 126; control untreated bio-replicate 2, 127N; control untreated bio-replicate 3, 127C; 4 h mock treatment bio-replicate 1, 128N; 4 h mock treatment bio-replicate 2, 128C; 4 h mock treatment bio-replicate 3, 129N; 4 h mock treatment bio-replicate 4, 129C; 4 h pilocarpine treatment bio-replicate 1, 130N; 4 h pilocarpine treatment bio-replicate 2, 130C; 4 h pilocarpine treatment bio-replicate 3, 131N; 4 h pilocarpine treatment bio-replicate 4, 131C. The 24 h TMT sample set consisted of: control untreated bio-replicate 1, 126; control untreated bio-replicate 2, 127N; control untreated bio-replicate 3, 127C; 24 h pilocarpine treatment bio-replicate 4, 128N; 24 h pilocarpine treatment bio-replicate 3, 128C; 24 h pilocarpine treatment bio-replicate 2, 129N; 24 h pilocarpine treatment bio-replicate 1, 129C; 24 h mock treatment bio-replicate 4, 130N; 24 h mock treatment bio-replicate 3, 130C; 24 h mock treatment bio-replicate 2, 131N; 24 h mock treatment bio-replicate 1, 131C (lot numbers TB266076 and TA265136). The two pooled samples were acidified with 100% formic acid to pH < 3 and the volume was reduced to 150 μL under vacuum centrifugation. The two samples were diluted to 2 mL with 0.1% trifluoracetic acid and desalted using solid phase extraction (Sep-Pak tC18 3cc Vac cartridge, Waters).

To obtain non-, mono- and multi-phosphorylated peptides from each timepoint, the samples were applied to the “TiSH” method of phosphopeptide enrichment.^77^ All recovered multi- and mono-phosphorylated enriched peptides were desalted, with multi-phosphorylated enriched samples ready for liquid chromatography tandem mass spectrometry (LC-MS/MS) analysis. The enriched mono-phosphorylated peptides were subjected to hydrophilic interaction chromatography (HILIC) for further fractionation prior to LC-MS/MS analysis. The mainly non-phosphorylated peptides which do not bind to titanium dioxide were collected from the first TiSH wash step. Approximately 3.5% of the 4 h and 24 h mainly non-phosphorylated peptides were desalted and fractionated by HILIC prior to LC-MS/MS analysis.

HILIC sample fractionation was completed using a Dionex UltiMate 3000 HPLC system (Thermo Fisher Scientific) controlled by Chromeleon 6.8 software with a TSKgel Amide-80 1 mm inside diameter x 250 mm long column (Tosoh Bioscience). Samples were injected in 150 μL in 90% acetonitrile, 9.9% water, 0.1% trifluoracetic acid (HILIC Buffer A) at a flow rate of 60 μL/min with buffer A at 100% for 10 min. The gradient was from 100% HILIC buffer A 100% to 40 % HILIC buffer B (99.9% water and 0.1% trifluoracetic acid) for 35 minutes at a flow rate of 50 μL/min, then to 80% buffer B for 3 minutes then back to 100% buffer A for 12 minutes. Sample fractions were collected in 60 s intervals into a 96 well plate using a Probot (LC Packings) controlled by μCarrier 2.0. Selected fractions were pooled based on the intensity of the ultraviolet absorbance at 214 nm resulting in seventeen phosphopeptide enriched fractions and twelve mainly non-phosphopeptide fractions. The mainly non-phosphorylated peptide HILIC fractionation was repeated and collected in 30 s fractions to make an additional set of twenty-seven fractions. Both 30 s and 60 s mainly non-phosphorylated peptide fractions were analysed. The fractions were dried under vacuum centrifugation and dissolved in 5.5 μL 0.1% formic acid for LC-MS/MS analysis.

#### TMT mass spectrometry

LC-MS/MS for all samples was completed with a Dionex UltiMate 3000 RSLC nano system and Q Exactive Plus hybrid quadrupole-orbitrap mass spectrometer (Thermo Fisher Scientific). The enriched multi-phosphorylated samples and HILIC fractions of the enriched non- and mono-phosphorylated samples were loaded directly onto a 300 × 0.075 mm column packed with ReproSil Pur C18 AQ 1.9 μm resin (Dr Maisch, Germany). A column oven (PRSO-V1, Sonation lab solutions, Germany) integrated with the nano flex ion source (Thermo Fisher Scientific) maintained the column at 50°C with an electrospray operating at 2.3 kV. The S lens radio frequency level was 50 and capillary temperature was 275°C. For MS analysis, 5 μL from all samples was injected. The reversed phase buffers were: A (0.1% formic acid in water) and B (0.1% formic acid, 90% acetonitrile, 9.9% water). There were two gradient variations used for MS acquisition. All enriched non- and mono phosphorylated fractions were subjected to a 110 min gradient beginning with sample loading at 99% buffer A and 1% buffer B separation for 25 min at 300 μL/min. The flow rate was then reduced to 250 μL/min in 30 s and the gradient was to 95% buffer A in 1 min, to 75% buffer A in 59 min, to 65% buffer A in 8 min, to 1% buffer A in 3 min and held at 1% buffer A for 2 mins before returning to 99% buffer A in 1 min and held for 10 min. For the multi phosphorylated samples the gradient was from 99% A to 95% buffer A in 6 min, to 72% buffer A in 116 min, to 65% buffer A in 10 min, to 1% buffer A in 3 min and held at 1% buffer A for 3 mins before returning to 99% buffer A in 2 min and held for 10 min. MS acquisition was performed throughout the 110 min and 180 min analysis.

Data-dependant acquisition LC-MS/MS was performed on all samples. In both the 110 min and 180 min methods, MS scans were at a resolution of 70,000 with an automatic gain control target of 1,000,000 for a maximum ion time of 100 ms from m/z 375 to 1500. Both the 110 min and 180 min methods had MS/MS scan resolution at 35,000 with an automatic gain control target of 200,000. The maximum ion time was 110 ms for the 180 min method and 115 ms for the 110 min method. The loop count was 12 for the 110 min method and 13 for the 180 min method. For both methods the isolation window was 1.2 m/z, the first mass was fixed at m/z 120 and the normalized collision energy was 34. Singly charged ions and those with charge >8 were excluded from MS/MS and dynamic exclusion was for 25 s in the 110 min method and 30 s in the 180 min method.

The raw LC-MS/MS data was processed with MaxQuant 1.6.7.0^71^ using the following settings. Variable modifications were oxidation of Met, acetylation of protein N-terminus, deamidation of Asn/Gln and phosphorylation of Ser/Thr/Tyr. Carbamidomethyl of Cys was a fixed modification. Digestion was set to Trypsin/P with a maximum of 3 missed cleavages. The inbuilt contaminants file was used as well as the *Mus musculus* reference proteome with canonical and isoform sequences downloaded Feb 21 2020. Also, a fast file containing the sequence of immunoglobulin G-binding protein A (UniProt accession: A0A0H3K686) was included. TMT-labelled protein A was added to selected samples to assess TMT ratio compression. The accession exists in the MaxQuant output but was removed in the post-MaxQuant analysis of the data. Minimum peptide length was 6 and maximum peptide mass was 6000 Da. Second peptides and dependent peptides searches were enabled. Peptide spectrum matching and protein false discovery rates were set to 0.01. Minimum score for modified peptides was 40. Modified peptides and counterpart non-modified peptides were not used in the calculation of protein level changes. Modified peptides or their non-modified counterparts were not used in the protein quantification. All other parameters were default. One razor plus unique peptide for proteins < 150 kDa and two razor plus unique peptides was required for proteins > 150 kDa for protein detection. The raw mass spectrometry files and MaxQuant results are available from the PRIDE repository and have the dataset identifier PXD038241.

#### Phosphopeptide enrichment for PRM samples

New pilocarpine and mock treated samples (four each) were produced and prepared for proteomics, as for the TMT analysis. Approximately 1 mg of peptide for each sample was desalted by solid phase extraction (Sep-Pak tC18 3cc Vac cartridge, Waters), dried and resuspended in water. 10% of the sample was reserved for PRM to determine protein levels. The remainder was used to enrich for phosphopeptides in each sample using titanium dioxide according to the first enrichment step of the TiSH method.^77^ Each sample was desalted and one third of the total was applied to PRM mass spectrometry.

#### PRM mass spectrometry

The samples for PRM analysis used the same LC-MS/MS equipment, column and reversed phase buffer system as for TMT. The enriched phosphopeptide samples, injected in 5 μL, were loaded in 99% buffer A for 25 min at 300 nL/min. The gradient, at 250 nL/min, was to 5% buffer B in 1 min, then to 25% buffer B in 97 min, to 35% buffer B in 3 min, to 99% buffer B in 1 min, held at 99% buffer B for 2 min, then to 99% buffer A in 1 min and held at 99% buffer A for 10 min as the flow rate increased to 275 nL/min. The peptide samples with no enrichment, injected in 3.5 μL, were loaded in 99% buffer A for 17.5 min at 300 nL/min. The gradient, at 250 nL/min, was to 6% buffer B in 1 min, then to 33% buffer B in 101.5 min, to 40% buffer B in 8 min, to 99% buffer B in 1 min, held at 99% buffer B for 2 min, then to 99% buffer A in 1 min and held at 99% buffer A for 10 min as the flow rate increased to 275 nL/min. Data-dependent acquisition runs were performed to determine phosphopeptide and non-phosphopeptide retention times. For the phosphopeptide PRM runs, the MS settings were as follows. For 140 min, full MS scans were performed at a resolution of 70,000 with automatic gain control target of 1,000,000, maximum scan time of 100 ms for the range 350 to 1500 m/z. The PRM scans were performed at a resolution of 35,000 with automatic gain control target of 3,000,000, maximum scan time of 175 ms, isolation window 0.9 m/z, normalised collision energy of 29 and loop count of 10. For non-phosphopeptides the MS settings were the same except the run was for 142 min, PRM resolution was 70,000, maximum ion time was 240 ms, isolation window was 0.7 m/z and normalised collision energy was 27. There were sixty-five phosphopeptide m/z targets, including four additional targets that were controls (not expected to change level according to TMT analysis). Due to multiple phosphorylation sites within each phosphopeptide, eluting at different retention times, the phosphorylation sites detected and quantified was greater than sixty-five. For non-phosphopeptides, two separate runs were performed. One run targeted seventy-four peptides belonging to eighteen proteins identified by TMT to have increased abundance and the other run targeted forty-nine peptides belonging to fourteen proteins identified by TMT to have decreased abundance. Both runs also targeted fourteen control peptides (not expected to change according to TMT results).

The targeted peptides were required to be unique in the mouse proteome at the gene level. The non-phosphopeptides were required to have no missed cleavages (trypsin specificity). Methionine was an allowed residue in only two of the one hundred and twenty-three target non-phosphopeptides. Target phosphopeptides had no restrictions on missed cleavages or amino acid residue content. Met-oxidised peptides were not targeted. Some Asn/Gln-deamidated phosphopeptides were targeted. One protein, collagen alpha-1(I), was represented by a single peptide. The remining proteins were represented by two to five peptides (average of 3.8 peptides/protein). The m/z target lists are available in Table S8.

#### Processing of PRM mass spectrometry data

The PRM runs were searched using Mascot 2.7.0 using the following settings for non-phosphopeptides. Enzyme specificity was trypsin and one missed cleavage was allowed. Carbamidomethylation of cysteine was a fixed modification. Peptide mass tolerance was 10 ppm. Fragment mass tolerance was 0.05 Da. The mouse proteome (download from UniProtKB Jan 2023 with canonical sequences) was searched. Phosphopeptides were searched in the same way except that two missed cleavages were allowed. Variable modifications were deamidation (Asn/Gln) and phosphorylation (Ser/Thr/Tyr). Additionally, the phosphopeptide PRM data was searched using MaxQuant 1.6.7.0 using the same settings as for the TMT data, with the following exceptions. No TMT labels were applied. The mouse proteome was downloaded from UniProtKB Oct 2023 with canonical and isoform sequences.

Quantitative data was obtained using Skyline 23.1.0.268. Each peptide was required to be detected with five transitions not including y_1_-y_2_ or b_1_-b_2_ ions, unless the peptide was shorter than 8 amino acid residues, in which case four transitions were sufficient and either y_2_ or b_2_ was allowed. The y_3_ and b_3_ ions were only allowed on peptides shorter than ten residues. Transitions had to be detected with less than 2.5 ppm accuracy. The phosphorylation site in peptides had to be localised by the Mascot or MaxQuant searches or manually by specific ions that rule out alternative sites. If no such ions were available, the site localisation was noted as ambiguous. The raw mass spectrometry files, search results and quantitative analysis files for the PRM analysis are available from the PRIDE repository and have the dataset identifier PXD047870.

#### Additional TMT MS data processing

The MaxQuant TMT data was further processed using methods and a ‘best annotation’ process described previously^21^ with a modification to the phosphopeptides retained for further analysis that are multi-phosphorylated. Briefly, all phosphorylation sites identified are first re-annotated and amino acid sequences obtained from the fasta file used for MaxQuant searching. Where multiple phosphorylation sites were detected on unique peptides or charge states, the median of the log_2_ transformed intensities was retained. This result was then considered a unique phosphopeptide for counting purposes. A difference to the previously described method is that all multi-phosphorylated peptides with a class 1 probability (i.e., a primary phosphorylated site) above 0.5 were retained if there was additional evidence of another class 1 site with a probability above 0.75 in at least one site from any experimental group. This allows retention of multi-site phosphorylated peptides and their intensities where evidence for the site combination existed amongst all experiments. Consistent with our previously described methods, all replicates were then globally normalised using quantile normalisation^78^ followed by imputation of missing data (each sample group was permitted 25% of missing data) using the knn nearest neighbour averaging method^79^ and corrected for unwanted technical sources of variation by application of surrogate variable analysis.^80^ After an initial analysis, the “MockInj_24_2” sample was removed as a significant outlier, assessed using the first 2 principle components of the normalised and corrected phosphopeptide intensities (mahalanobis distance p-value = 0.0001).^81^ Data were then remapped as described above (without the “MockInj_24_2” sample) before further analysis. For differential phosphorylation and protein analysis, data were fit with a generalized linear model with Bayes shrinkage implemented in limma version 3.36.5^73^ comparing the pilocarpine and mock injections for each matched timepoint and for each mock injection versus the pooled controls at 0 minutes.

#### Additional PRM MS data processing

For both protein and phosphorylation PRM results, intensities were log2 transformed, globally normalised so that each sample had the same median value and fit with a generalized linear model with Bayes shrinkage implemented in limma version 3.36.5^73^ comparing the pilocarpine and mock injections for each matched timepoint.

#### KinSwing

Protein kinase activity was predicted with KinSwingR version 1.1.4 (described here:^21^ and implemented/available on R/BioConductor with additional implementation of significance scoring of kinase activity: http://bioconductor.org/packages/KinSwingR/). Briefly, KinSwing predicts kinase activity by first modelling kinase matches (position weight matrices of known mammalian kinase:substrate sequences) to each phosphorylated peptide. Kinase activity is then inferred using a network theoretic approach, where nodes represent kinases and edges represent modelled kinase:substrate interactions. Local network connectivity for each kinase is determined as the absolute proportion of positive and negative peptides that have significant fold changes (P ≤ 0.05), weighted by the number of edges and number of substrate sequences using to build the kinase model. Finally, the weighted kinase score is z-transformed to enable comparison between different conditions.

The KinSwingR predictions of Ser/Thr activity were compared to the changes in phosphorylation status of known protein kinase substrates in the data by first downloading the known substrates from PhosphoSitePlus^27^ (downloaded November 2 2020). For each protein kinase identified by KinSwingR, the average significant phosphopeptide intensity for all known substrates in our data was calculated. A minimum of four substrates was required. Kinase-substrate relationships from all mammals were allowed if the gene name and the sequence within four residues either side of the phosphorylation site was identical. All duplicate combinations of genes and sequence values from PhosphoSitePlus using these criteria were discarded. Multi-site phosphorylated peptides were not simplified or discarded but kept throughout all analyses. The first sequence window for the first phosphorylation site was assigned the quantitative value for the phosphopeptide in sequence analyses. In displaying data for Ser and Thr or Tyr protein kinases, those protein kinases with specificity for all three residues were repeated in each display. Kinase specificity was determined using the “Ser/Thr protein kinase activity” or “Tyr protein kinase activity” term from UniProt. Pseudo-kinases were allowed when displaying protein kinase log_2_ fold change data. Membership of various other enriched gene ontology terms was determined using QuickGO.

#### Gene ontology enrichment

The significantly regulated phosphopeptides for the four comparisons designated P/U(4 h), P/U(24 h), D/U(4 h) and D/U(24 h) were divided into those that increased or decreased abundance, resulting in eight sets. The phosphopeptides were ranked from most significant to least significant and then from largest to smallest quantitative value. The significance ranking value and quantitative ranking value was summed for each phosphopeptide to produce a final combined rank. The gene for each phosphopeptide was extracted to produce a ranked gene list. Redundant gene names were discarded, retaining the highest ranked gene name for each phosphopeptide. The ranked gene lists were submitted to gProfiler^24^ using the ‘multi-query’ and ‘ordered query’ options. Note that an ordered query is an iterative process that may not use the entire ranked gene list to obtain significance,^24^ weighting the output toward the higher ranking genes. The organism was set to *Mus musculus* (version: GRCm38.p6). A custom background was used which consisted of all the genes represented by the proteins detected in both the proteomics and phosphoproteomics. Genes without annotation were included in the background. The following data sources and versions were used: GO Molecular Function release June 1 2020, GO cellular component release June 1 2020, GO Biological Process release June 1 2020, KEGG release July 6 2020, Reactome annotations ensemble classes July 7 2020 and WikiPathways version June 10 2020.

Significant protein values were also divided into groups of increased or decreased abundance and ranked by both significance and by the magnitude of the quantitative values. For protein groups mapping to multiple genes, the first gene (ranked by MaxQuant rules) was kept and the remaining genes discarded. Redundant genes were also removed before submission to gProfiler using the same settings as for phosphopeptides. The list of significant terms was simplified using REVIGO.^75^ The full output from gProfiler is supplied Table S3.

In a separate analysis of persistently regulated phosphopeptides and proteins, the log_2_ fold change was required to be significant for both 4 and 24 h for inclusion. The same procedure for GO enrichment was followed except that the background was the subset of annotated genes in the *Mus musculus* genome (version GRCm39), the threshold for significance was set to 0.00001, the term size was limited to 400 genes and the data sources were newer. We used GO Molecular Function, GO cellular component and GO Biological Process released March 22 2022.

#### Phosphorylation site function analysis

The phosphorylation sites that are known to induce or inhibit a particular function were obtained from PhosphoSitePlus^27^ (downloaded August 28 2020). We sequence matched the PhosphoSitePlus data to our data requiring an identical gene name and sequence within four residues either side of the phosphorylation site. Sequences from all mammals were allowed. Multi-site phosphopeptides were matched using the sequence window of the first phosphorylation site. If this caused a redundant match, the quantitative value with the largest magnitude was kept and the remainder discarded.

#### Immunolabelling of hippocampal slices

4 h post induction of SE, brain hemispheres were harvested and acutely sliced using a vibrating microtome (Microm HM 650 V, ThermoScientific) into 400 μm coronal sections in cold HEPES-buffered Krebs-Ringer solution (120 mM NaCl; 5 mM KCl; 2 mM CaCl2; 1 mM MgCl2; 25 mM NaHCO3; 5.5 mM HEPES; 1.1 mM D-Glucose; pH 7.4), then fixed in 4% paraformaldehyde overnight at 4°C. The sections were embedded in 3% agar (Sigma) before re-slicing into 50 μm coronal slices. After washing in TBS (20 mM Tris; 150 mM NaCl; pH 7.6), free floating slices were incubated overnight at 4°C with anti-GluA1 pS863 (PA5-104896, Invitrogen, 1:200) in TBS with 0.1% Triton X-100. Immunolabelled slices were washed before subsequently incubating with rabbit secondary antibody conjugated with Alexa Fluor 568 (A11011, Invitrogen) and Hoechst 33342 (Invitrogen, 1:1000) in TBS with 0.1% Triton X-100 for 3 hr at 35°C. Slices were washed then mounted with Fluoromount™ Aqueous Mounting Medium (Sigma) and imaged using a Leica SP8 point scanning microscope and analysed using ImageJ (National Institutes of Health).

### Quantification and statistical analysis

For differential phosphorylation and protein analysis by TMT or PRM mass spectrometry, statistical significance was determined using a moderated t-test within limma version 3.36.5.^73^ Then, multiple comparison correction was performed (adjustment of P values) using the Benjamini and Hochberg method was applied. An adjusted P value of <0.05 was considered significant. Phosphopeptides that exhibited underlying changes in protein were excluded from the figures and further analyses. Phosphopeptide data with no corresponding protein level data was kept, representing approximately one third of the detected phosphopeptides (Figure S2C). Averaged mass spectrometry data was clustered using Morpheus (https://software.broadinstitute.org/morpheus/). Protein or phosphopeptide data was hierarchically clustered using Euclidian distance with a complete linkage.

For KinSwing,^21^ significance of the kinase activity score ${swing}_{k}$ was determined by permutating kinase node labels, K, to substrates, S, of the total network, $M_{ks}$. Thereby the probability of observing ${swing}_{k}$ is conditional on this permuted reference distribution, of size, N (1,000 permutations), and is computed for each tail of the distribution, positive and negative ${swing}_{k}$ scores. For gene ontology enrichment, the significance threshold was P < 0.05 and the gProfiler algorithm for adjustment of multiple hypotheses was applied.^82^ The Pearson correlation coefficient for the comparison of TMT and PRM data was determined using GraphPad Prism 9.02.

## Supplemental information

Document S1. Figures S1–S8

Table S1. Phosphopeptide intensities, log2 fold changes and probabilities, related to Figures 1, 2, 3, 4, and 5 A.) Log_2_ fold changes, probabilities and ranks for all comparisons. B.) Log_2_ intensities at 4 h. C.) Log_2_ intensities at 24 h. D.) Log_2_ fold changes for persistently regulated phosphopeptides.

Table S2. Protein log2 fold changes, probabilities and raw data, related to Figure 6 A.) Log_2_ fold change comparisons, probabilities and number of supporting peptides for both 4 and 24 h. B.) Raw MaxQuant protein output.

Table S3. Gene ontology enrichment results for phosphopeptide data, related to Figures 1, 3, 4, and 5 A.) The gProfiler combined output for all comparisons. B.) Detailed gProfiler output for P/M(4 h) increased phosphorylation. C.) P/M(4 h) decreased phosphorylation. D.) P/M(24 h) increased phosphorylation. E.) P/M(24 h) decreased phosphorylation. F.) D/U(24 h) increased phosphorylation. G.) D/U(24 h) decreased phosphorylation. H.) D/U(24 h) increased phosphorylation. I.) D/U(24 h) decreased phosphorylation. J.) Persistently increased phosphorylation, for both P/M(4 h) and P/M(24 h). K.) Persistently decreased phosphorylation, for both P/M(4 h) and P/M(24 h).

Table S4. Inferred protein kinase activity predictions by KinSwing, related to Figures 3, 4, and 5 A.) Predictions at 4 and 24 h for the P/M(4 h) and P/M(24 h) comparisons. B.) Predictions at 4 and 24 h for the D/U(4 h) and D/U(24 h) comparisons.

Table S5. Gene ontology enrichment results for protein data, related to Figure 6 A.) The gProfiler combined output for all comparisons. B.) P/M(4 h) decreased protein abundance. C.) P/M(24 h) increased protein abundance. D.) P/M(24 h) decreased protein abundance. E.) D/U(24 h) decreased protein abundance.

Table S6. Known protein markers of epileptogenesis up to 24 h, related to Figure 6 A.) Proteins found previously to change abundance in the first 24 h of epileptogenesis in various models, organisms, brain regions and detected via various analysis methods.

Table S7. Results for the parallel reaction monitoring analysis, related to Figure 7 A.) Log_2_ fold changes and probabilities for the detected phosphopeptides. B.) Log_2_ fold changes and probabilities for the detected proteins.

Table S8. Target list for the parallel reaction monitoring analysis, related to Figure 7 A.) The m/z values and elution time windows for the targeted phosphopeptides. B.) The m/z values and elution time windows for the peptides representing targeted proteins.
