# Supplementary material for: Multiomics of early epileptogenesis in mice reveals phosphorylation and dephosphorylation-directed growth and synaptic weakening
Source: iScience. 2024 Mar 19;27(4):109534. doi: 10.1016/j.isci.2024.109534 (PMC11005001; doi:10.1016/j.isci.2024.109534)
Supplement: Document S1. Figures S1–S8 [file mmc1.pdf]

iScience, Volume *27*

Supplemental information

Multiomics of early epileptogenesis in mice reveals phosphorylation and dephosphorylation-directed growth and synaptic weakening

Mariella Hurtado Silva, Ashley J. van Waardenberg, Aya Mostafa, Susanne Schoch, Dirk Dietrich, and Mark E. Graham

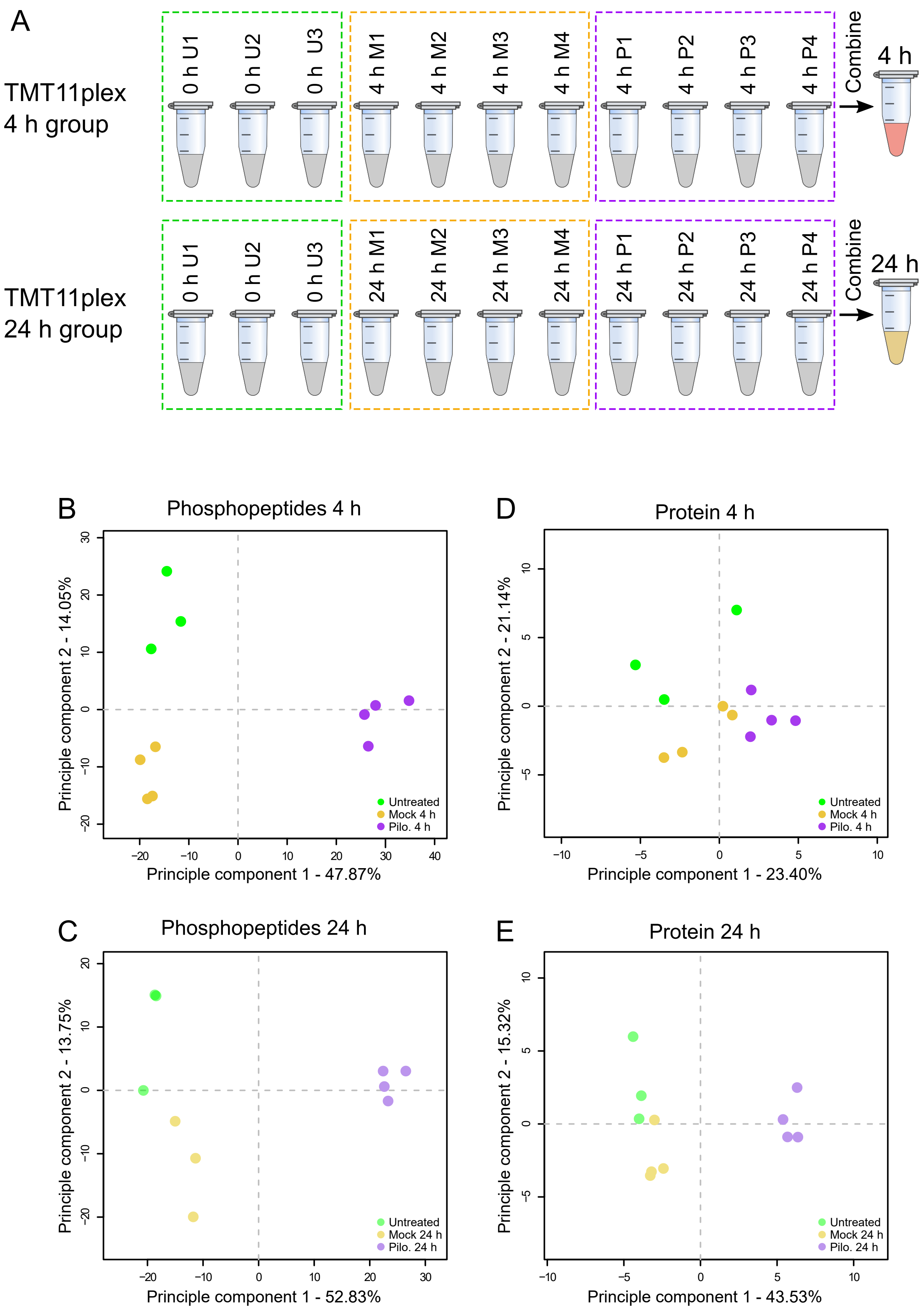

Figure S1. Tandem mass tag labelling scheme and principal component analyses, related to Figure 1. TMT11plex labelling scheme. Three untreated controls from the zero time point, four mock injected and four pilocarpine samples collected at the 4 h time point were in one group. The same three untreated controls from the first group, four mock injected and four pilocarpine samples collected at the 24 h time point were in the second group. The principal component analysis of the normalised and batch corrected sample for (B) the phosphoproteome data at 4 h, (C) 24 h, (D) the proteome data at 4 h and (E) 24 h.

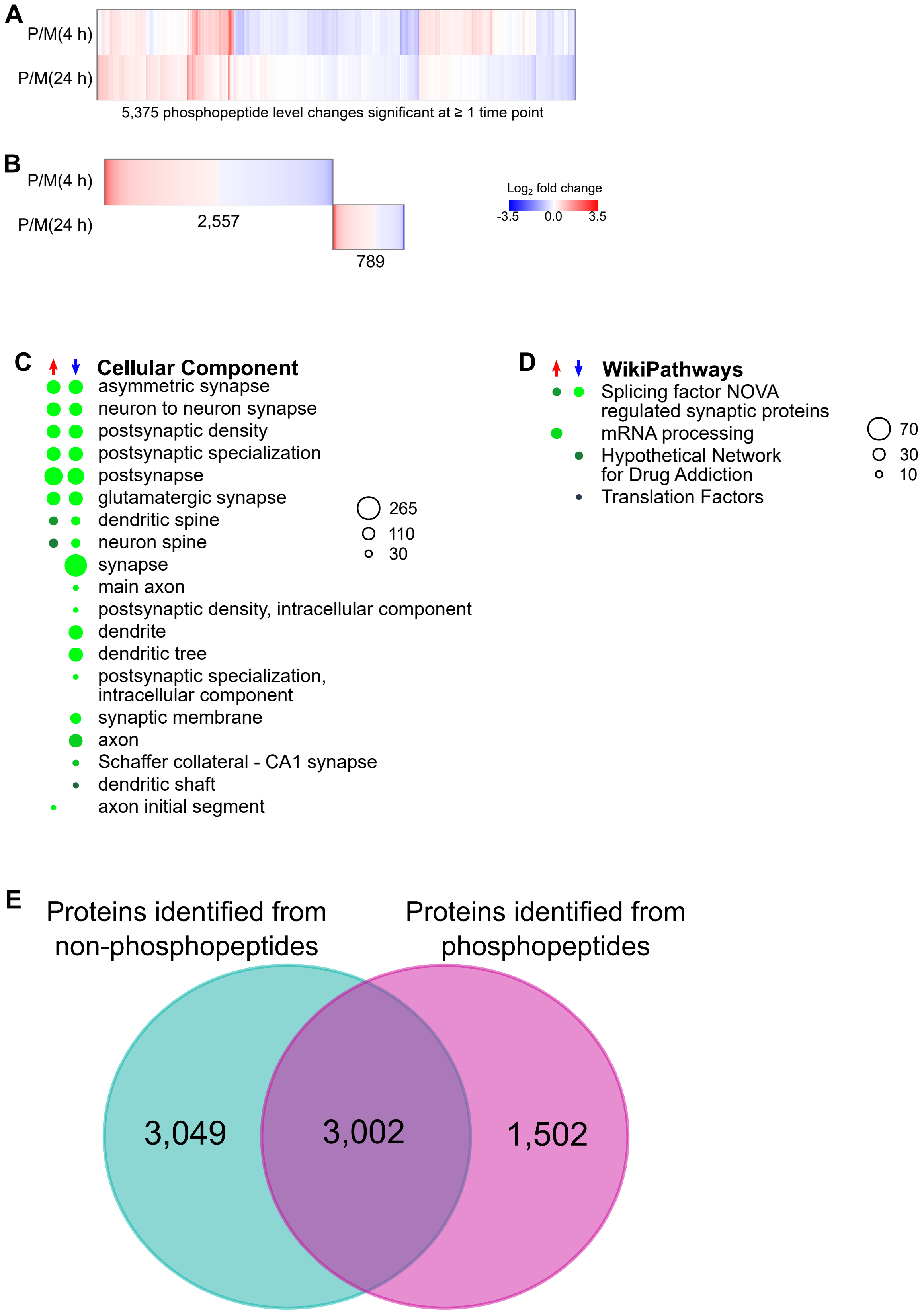

Figure S2. Phosphoproteome heat map, Venn diagram of the detected proteins and 4 h phosphoproteome gene ontology terms, related to Figure 1.
(A) Heat map of phosphorylate site changes detected at one or more time points. (B) Phosphorylation site changes detected at a single time point. (C) Venn diagram of proteins detected from non-phosphopeptides and phosphopeptides. Proteins with redundant gene names were removed before calculating the total. Enriched (D) cellular component and (E) WikiPathways terms for the phosphoproteome data at 4 h.

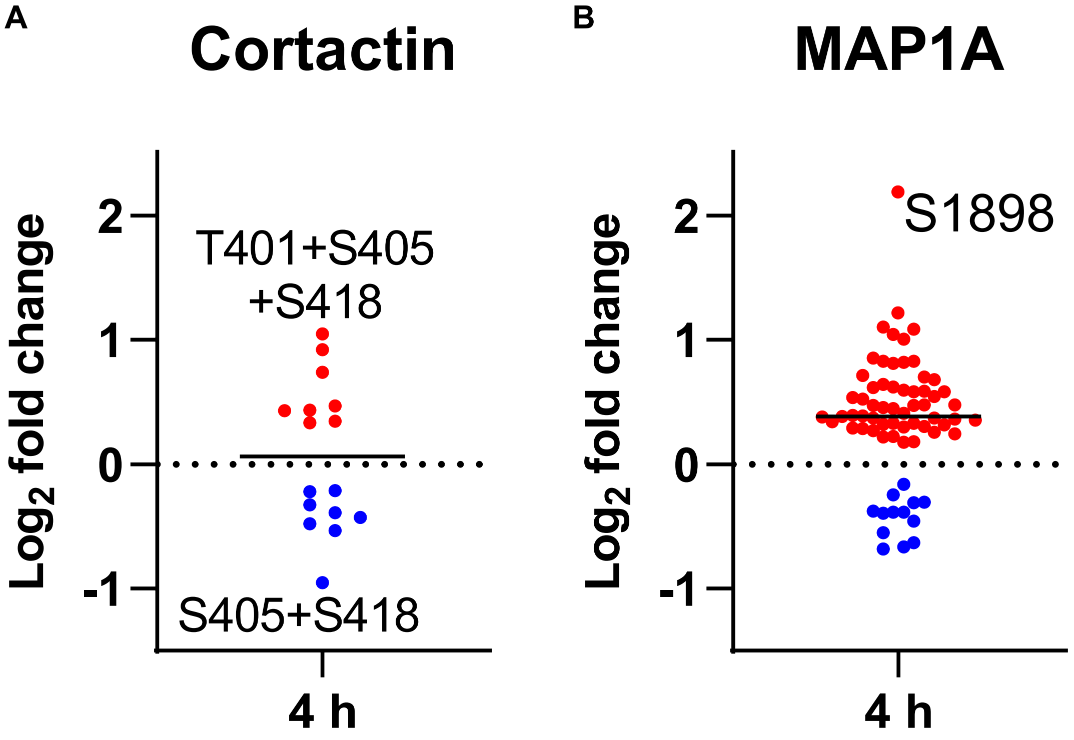

Figure S3. Examples of multisite phosphorylation and median log2 fold change for two postsynaptic proteins, related to Figure 1.
Plots of phosphopeptide log2 fold changes for (A) cortactin and (B) MAP1A at 4 h. Each dot represents a significant phosphorylation site change. The median value is shown by the black line.

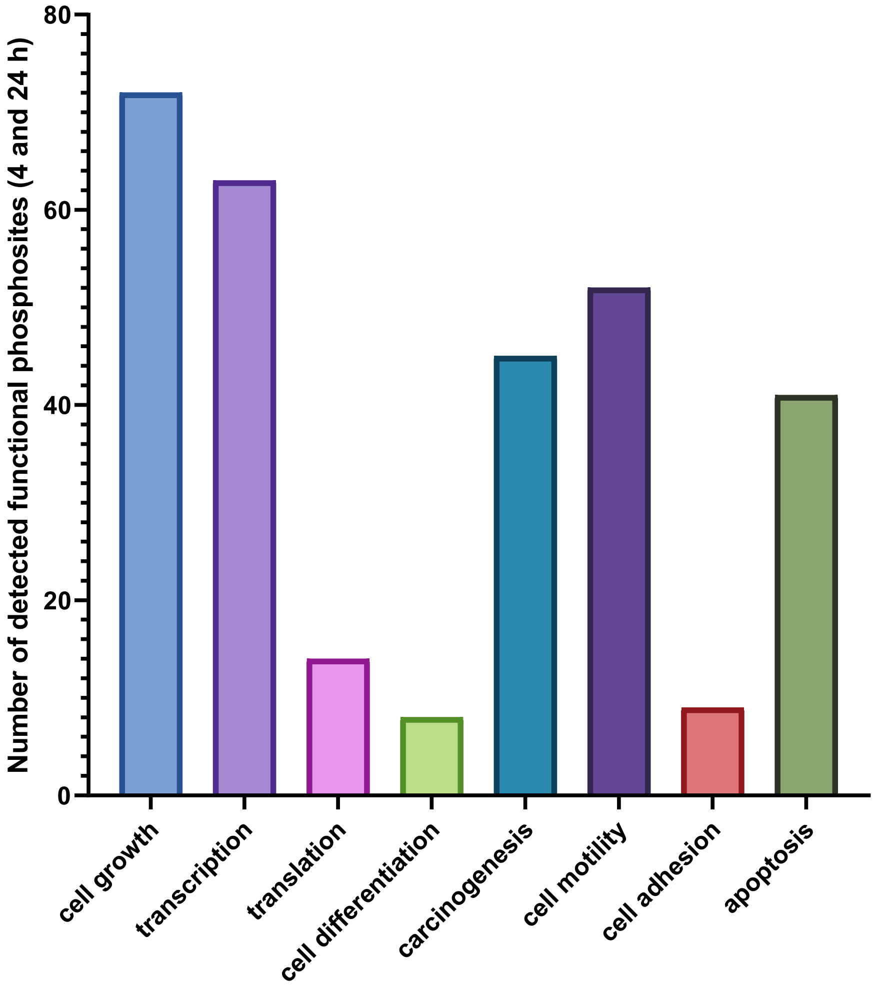

Figure S4. The total number of detected functional phosphorylation sites, according to PhosphoSitePlus, for various cellular functions at both 4 and 24 h after SE, related to Figure 2.

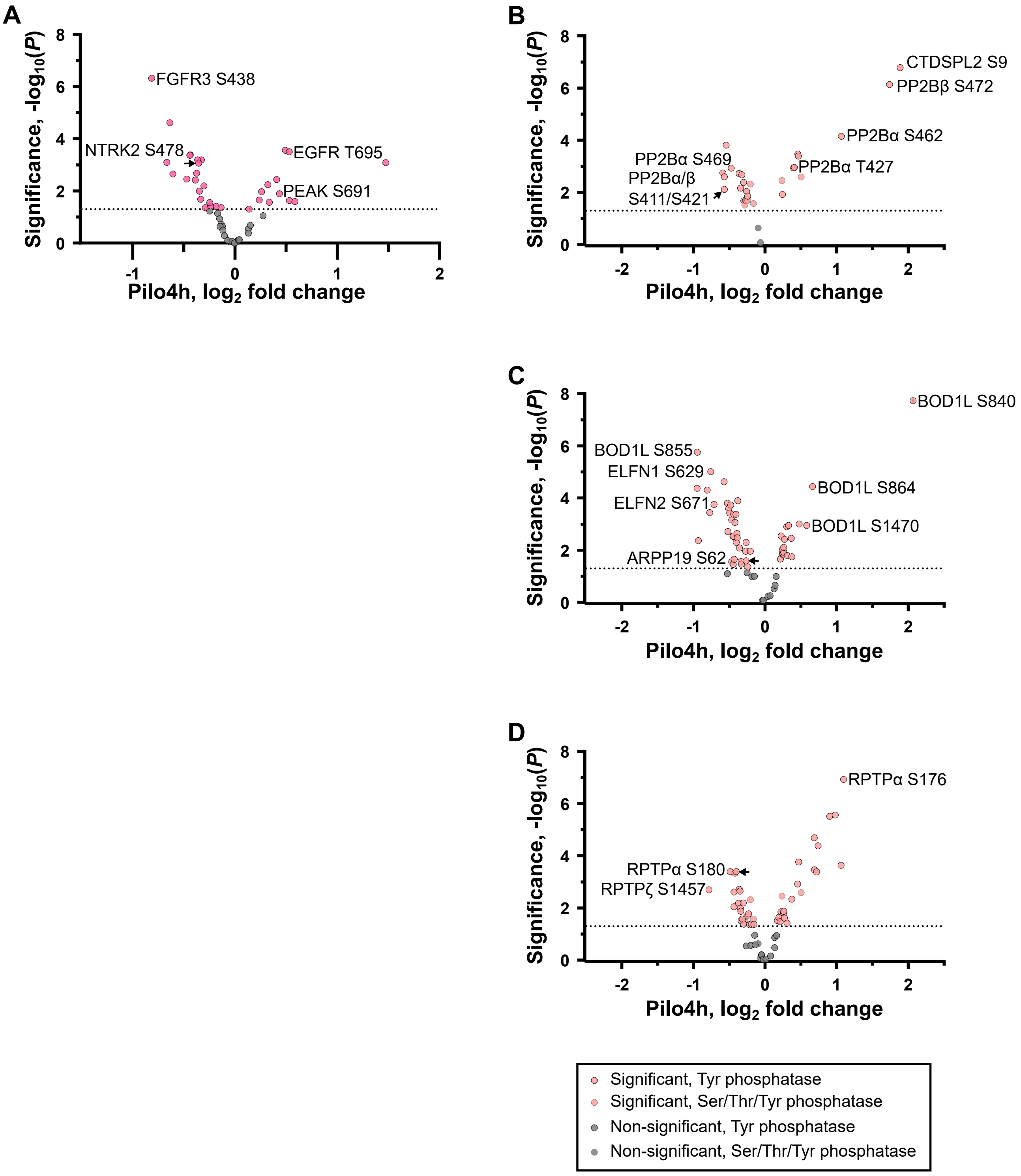

Figure S5. Volcano plots for protein kinase, phosphatases and phosphatase regulators at 4 h after SE, related to Figure 2.

Volcano plot of the log2 fold changes for phosphopeptides occurring on (A) Tyr protein kinases (B) (A) Ser/Thr protein phosphatases, (C) Ser/Thr protein phosphatase regulatory proteins and (D) Tyr protein phosphatases perturbed by pilocarpine at 4 h after SE. Protein phosphatases with specificity for phospho -Ser, Thr and Tyr are shown in both (B) and (C).

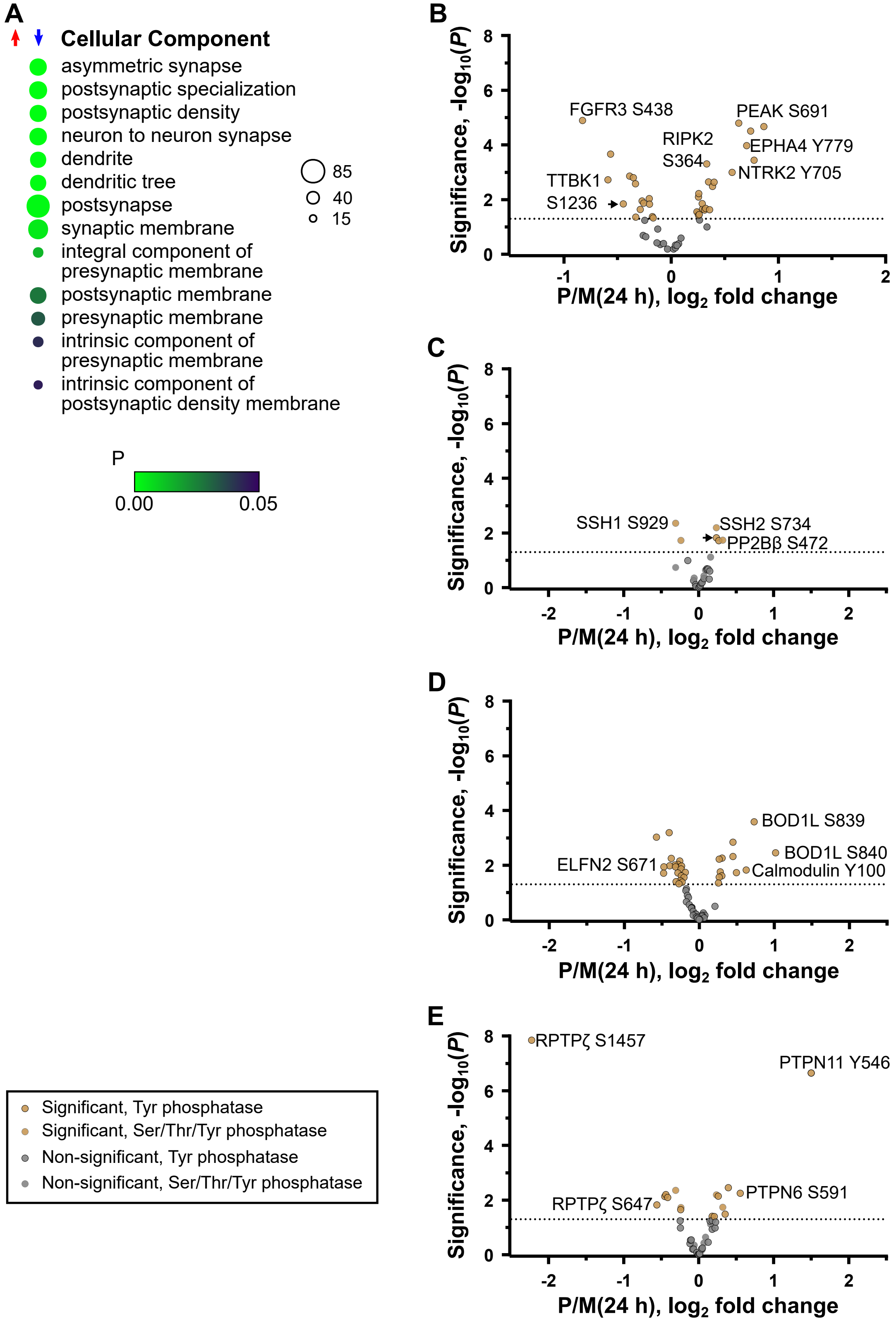

Figure S6. Gene ontology enrichment and volcano plots for protein kinase, phosphatases and phosphatase regulators at 24 h after SE, related to Figure 4.

(A) Enriched cellular component terms for the phosphoproteome data at 24 h. Volcano plot of the log2 fold changes for phosphopeptides occurring on (B) Tyr protein kinases (C) (A) Ser/Thr protein phosphatases, (D) Ser/Thr protein phosphatase regulatory proteins and (E) Tyr protein phosphatases perturbed by pilocarpine at 24 h after SE. Protein phosphatases with specificity for phospho-Ser, Thr and Tyr are shown in both (C) and (D).

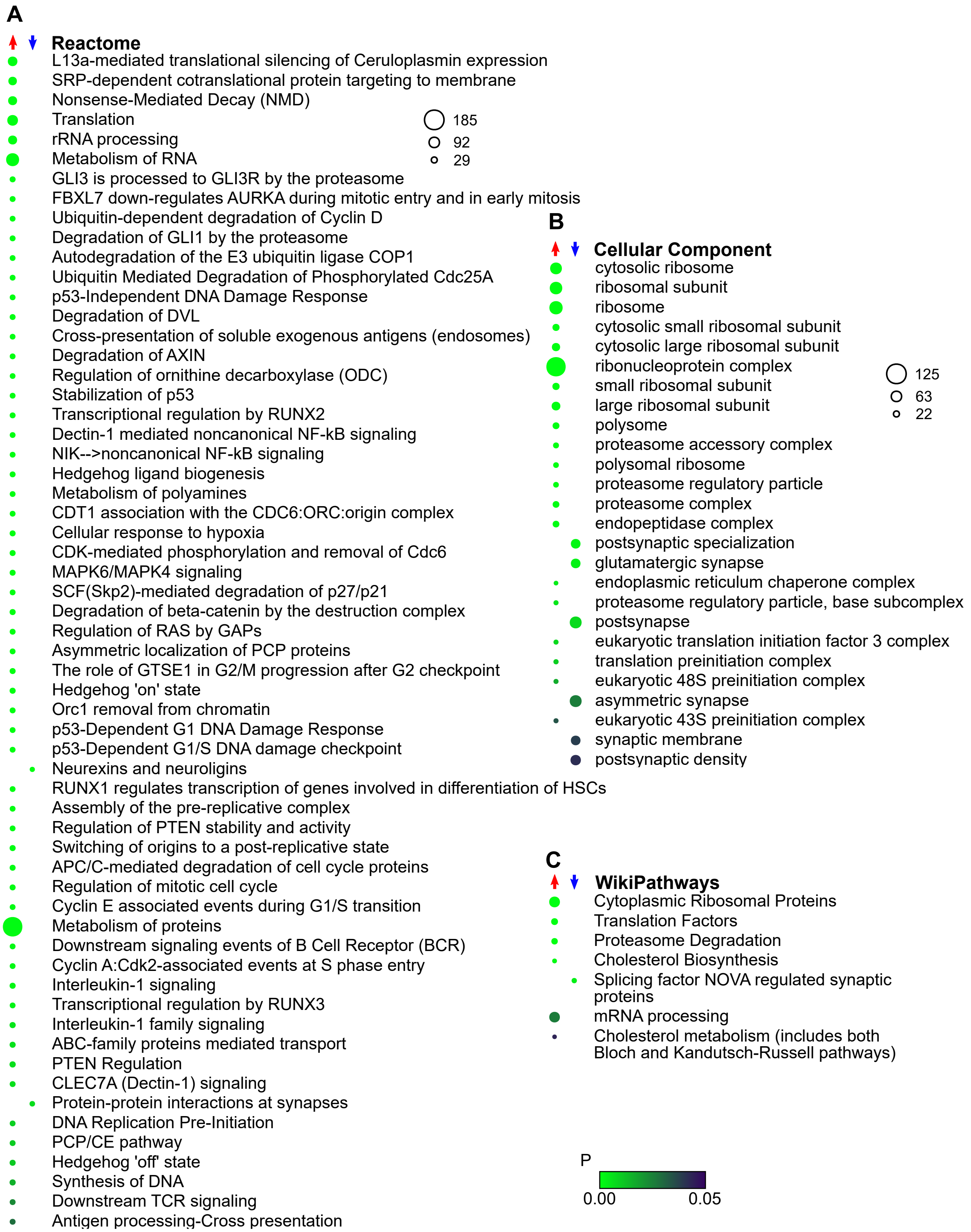

Figure S7. Reactome, cellular component and WikiPathways terms enriched by analysis of the proteome at 24 h after SE, related to Figure 6.

Enriched (A) Reactome, (B) cellular component and (C) WikiPathways terms for proteins with significantly different levels at 24 h after SE. A scale bar for probability is shown. The size of the circle represents the number of genes enriched.

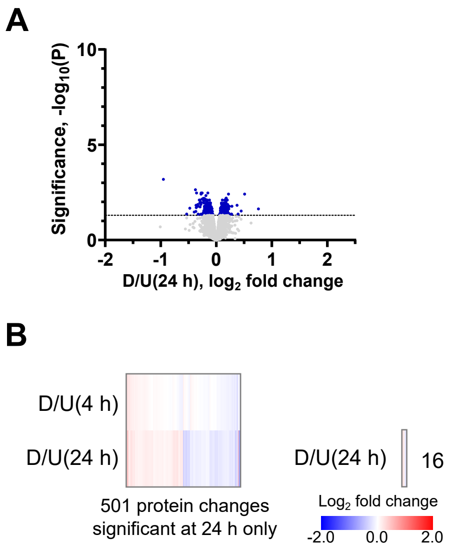

C

Biological Process

- mitochondrial respiratory chain complex assembly

D

Cellular Component

- inner mitochondrial membrane protein complex
- mitochondrial protein complex
- prefoldin complex
- mitochondrial respirasome
- respiratory chain complex I
- NADH dehydrogenase complex
- mitochondrial respiratory chain complex I
- respiratory chain complex
- respirasome

E

KEGG

- Oxidative phosphorylation

F

Reactome

- Respiratory electron transport, ATP synthesis by chemiosmotic coupling, and heat production by uncoupling proteins.
- Complex I biogenesis

G

WikiPathways

- Electron Transport Chain
- Oxidative phosphorylation
- Cholesterol Biosynthesis

Figure S8. Protein changes and gene ontology enrichment after diazepam treatment at 24 h, related to Figure 6.
(A) Volcano plot of the significantly regulated proteins at 24 h. Light grey dots are below the threshold for significance. (B) Heat map of hierarchically clustered log2 fold changes for protein groups significantly changing at 24 h aligned to values at 4 h. There were no significant changes at 4 h. Enriched (C) biological process (D) cellular component, (E) KEGG, (F) Reactome and (G) WikiPathways terms for protein changes at 24 h
